# Prolonged Siberian heat of 2020 almost impossible without human influence

**DOI:** 10.1007/s10584-021-03052-w

**Published:** 2021-05-06

**Authors:** Andrew Ciavarella, Daniel Cotterill, Peter Stott, Sarah Kew, Sjoukje Philip, Geert Jan van Oldenborgh, Amalie Skålevåg, Philip Lorenz, Yoann Robin, Friederike Otto, Mathias Hauser, Sonia I. Seneviratne, Flavio Lehner, Olga Zolina

**Affiliations:** 1 Met Office Hadley Centre, FitzRoy Road, Exeter, EX1 3PB UK; 2 Royal Netherlands Meteorological Institute (KNMI), De Bilt, The Netherlands; 3 Deutscher Wetterdienst (DWD), Güterfelder Damm 87-91, 14532 Stahnsdorf, Germany; 4 Météo France, Paris, France; 5 Environmental Change Institute, University of Oxford, Oxford, OX UK; 6 Institute for Atmospheric and Climate Science, ETH Zürich, Zürich, Switzerland; 7 IGE/UGA, Grenoble, France; 8 P.P.Shirshov Institute of Oceanology, Moscow, Russia

**Keywords:** Extreme Event Attribution, Heatwave, Siberia, Extremes, Multi-model, Rapid attribution

## Abstract

Over the first half of 2020, Siberia experienced the warmest period from January to June since records began and on the 20th of June the weather station at Verkhoyansk reported 38 °C, the highest daily maximum temperature recorded north of the Arctic Circle. We present a multi-model, multi-method analysis on how anthropogenic climate change affected the probability of these events occurring using both observational datasets and a large collection of climate models, including state-of-the-art higher-resolution simulations designed for attribution and many from the latest generation of coupled ocean-atmosphere models, CMIP6. Conscious that the impacts of heatwaves can span large differences in spatial and temporal scales, we focus on two measures of the extreme Siberian heat of 2020: January to June mean temperatures over a large Siberian region and maximum daily temperatures in the vicinity of the town of Verkhoyansk. We show that human-induced climate change has dramatically increased the probability of occurrence and magnitude of extremes in both of these (with lower confidence for the probability for Verkhoyansk) and that without human influence the temperatures widely experienced in Siberia in the first half of 2020 would have been practically impossible.

**Supplementary Information:**

The online version contains supplementary material available at 10.1007/s10584-021-03052-w.

## Introduction

Since the beginning of 2020, anomalously high temperatures were repeatedly reported in Siberia. For instance, on 17 June 2020, the Guardian reported that Russia as a whole had experienced record high temperatures in 2020, with the average from January to May being 5.3 °C above the 1951–1980 average (Guardian 2020) and contributing to January to May globally averaged temperatures ranking 2nd warmest on record (Met Office global temperature 2020). On the 23rd of June, the World Meteorological Organization (WMO) announced that it was ‘seeking to verify a reported new record temperature north of the Arctic Circle [of] 38°C on 20^th^ June in the Russian town of Verkhoyansk amid a prolonged Siberian heatwave and increase in wildfire activity’ (WMO 2020). Notably, this event happened in the month prior to that of the climatologically expected peak daily maxima. Subsequently, numerous media (newspapers, television, radio) reported on the event as well as on the Siberian heat anomaly persisting since early 2020. The 20th of June Arctic temperature record was then confirmed on the 30th of June by Russia’s meteorological service Roshydromet (WMO Twitter). We noted that this temperature is not reproduced by the (lower than station resolution) ERA5 reanalysis, which reaches 32.9 °C on the 21st of June in the vicinity of Verkhoyansk but which is still a record in this dataset, supporting the exceptional nature of the heat locally over this period. This article is based on a rapid attribution study performed by the World Weather Attribution consortium and the scientific report underlying that study.

**Fig. 1:**
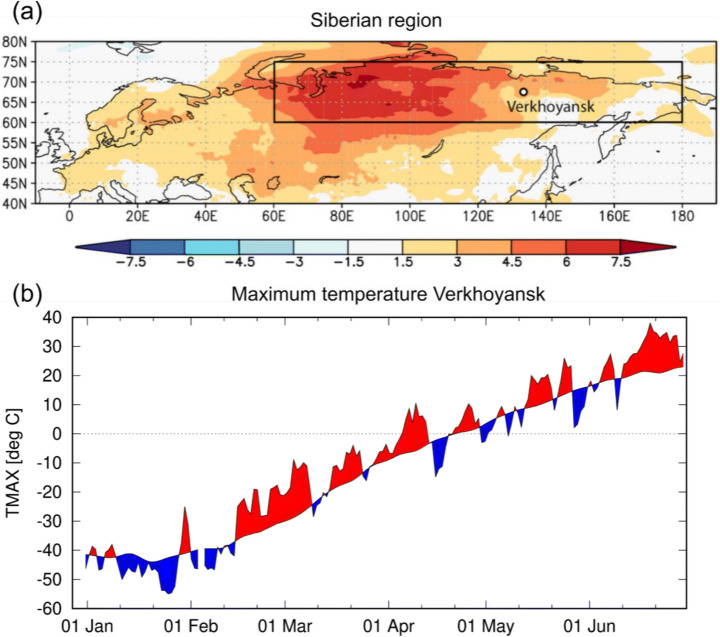
Extreme Siberian heat of 2020. **a** ERA5 near-surface temperature (T2m) anomalies (with respect to 1981–2010) [°C] for January–June 2020. The region used in the study (black box) 60°N–75°N, 60°E–180°E saw an anomaly of +4.5C w.r.t. 1981–2010. The location of Verkhoyansk is also shown. **b** Daily maximum temperature (TX) observations [°C] from January to June 2020 at station Verkhoyansk with positive and negative departures from the 1981–2010 climatological mean shaded red and blue, respectively. TX peaks at 38 °C on the 20th of June

Here, we investigate the role of human-induced climate change in the likelihood and intensity of both of these events: the persistent warm anomalies across Siberia (here defined as 60°N–75°N, 60°E–180°E) from January to June 2020 (black box in Fig. 1 (a)) and the reported record temperature of 38 °C at Verkhoyansk (67.55°N, 133.38°E) on the 20th of June (Fig. 1 (a)). Both of these event definitions are chosen primarily to relate to the impacts of the extreme heat on the ecosystem and human health. Changes in likelihood are expressed through probability ratios (PR), i.e. the change in probability of occurrence of an event at least as extreme as that observed, calculated as the ratio of the threshold exceedance probabilities at two different times. An alternative way to analyse the event is to consider intensity changes, representing the change in temperature of the event for a fixed probability.

While the record temperature north of the Arctic circle on the 20th of June made headlines, impacts potentially linked directly or in part to the extreme heat have been widespread. Persistent and unusually many wildfires have been observed. About 7900 mile^2^ of Siberian territory had burned this year as of the 25th of June, compared to a total of 6800 mile^2^ as of the same date a year ago, according to official data (New York Times 2020a; National Geographic 2020). These fires led to a release of 56 megatons of CO2 in June 2020 (COPERNICUS wildfires 2020), more than the yearly CO2 emissions of some countries (e.g. Switzerland (Global Carbon Atlas)). High temperatures and also the dry conditions in the first 6 months probably exacerbated these fires. Further impacts include health impacts on the population (New York Times 2020b) and the melting of permafrost which led to high damages, including environmental pollution: ‘A fuel tank near the isolated Arctic mining city of Norilsk burst in late May after sinking into permafrost that had stood firm for years but gave way during a warm spring, officials said. It released about 150,000 barrels of diesel into a river’ (New York Times 2020b).

It is important to highlight that the meteorological extremes assessed here only represent part of one component of these impacts, the hazard, whereas the impacts also depend strongly on exposure and vulnerability, as well as other climatological components such as the duration of extreme heat.

The high temperatures in Siberia in January–April were associated with below-normal surface pressure over the Arctic Ocean, extending south into northern Siberia (Fig. S1). In this season, low pressure is associated with milder temperatures as it inhibits the clear skies of the Siberian High that cause strong longwave radiative cooling from the snow. This pattern persisted, being very strong in January–March and less strong in April. It also supports direct advection of relatively warmer and moister air from lower latitudes. A detailed analysis of factors that can lead to such situations is provided in Wu and Chen (2020). In May–June, the opposite connection holds: higher pressure leads to more sunshine, which increases the temperature. We indeed find high sea-level pressure during June in the study area. Persistence of the high-temperature anomalies was likely enhanced during May and June due to earlier snowmelt (Fig. S2). The bare soil absorbs more solar radiation and hence can cause higher temperatures.

The synoptic development that led to the record temperatures in Verkhoyansk (Fig. 1 (b)) was initially associated with the blocking of the subpolar jet by a persistent low over Central Siberia. The blocking developed on 6–8 June 2020, resulting in a moderate ridge over eastern Siberia (east of Verkhoyansk). This pattern likely originated from a cut-off of the North Pacific anticyclone and preconditioned the high-temperature anomaly in the second half of June. This pattern was characterized by geopotential height at 500 mbar (z500) exceeding 5580 gpm and mean sea-level pressure (MSLP) peaking at 1013–1014 hPa. Notably, daily maximum temperatures increased locally to 27.8–28.0 °C on the 8th of June. After the 12th of June, this high-pressure centre expanded over much of eastern Siberia (including Verkhoyansk) while local temperature experienced a short-term decrease. Starting from the 16th–17th of June, this high-pressure centre was under the influence of the intense transport of the tropical air masses associated with propagation of the tropical high-pressure ridge from the south northeastward, potentially associated with the impact of the spring-early summer Asian monsoon (e.g. Choi and Ahn 2019). This resulted in sustained high-pressure centred east of Verkhoyansk with maximum z500 exceeding 5800 gpm and advection of very hot air from the south. Mean daily temperatures between the 17th and 27th of June exceeded 10 °C above the 1960–2010 norm and peaked at 13.7 °C above normal. Additionally, the period 10th to 30th of June was an exceptional dry spell, exceeding the 99.9th percentile of dry spell duration estimated according to the methodology of Zolina et al. (2013).

**Fig. 2:**
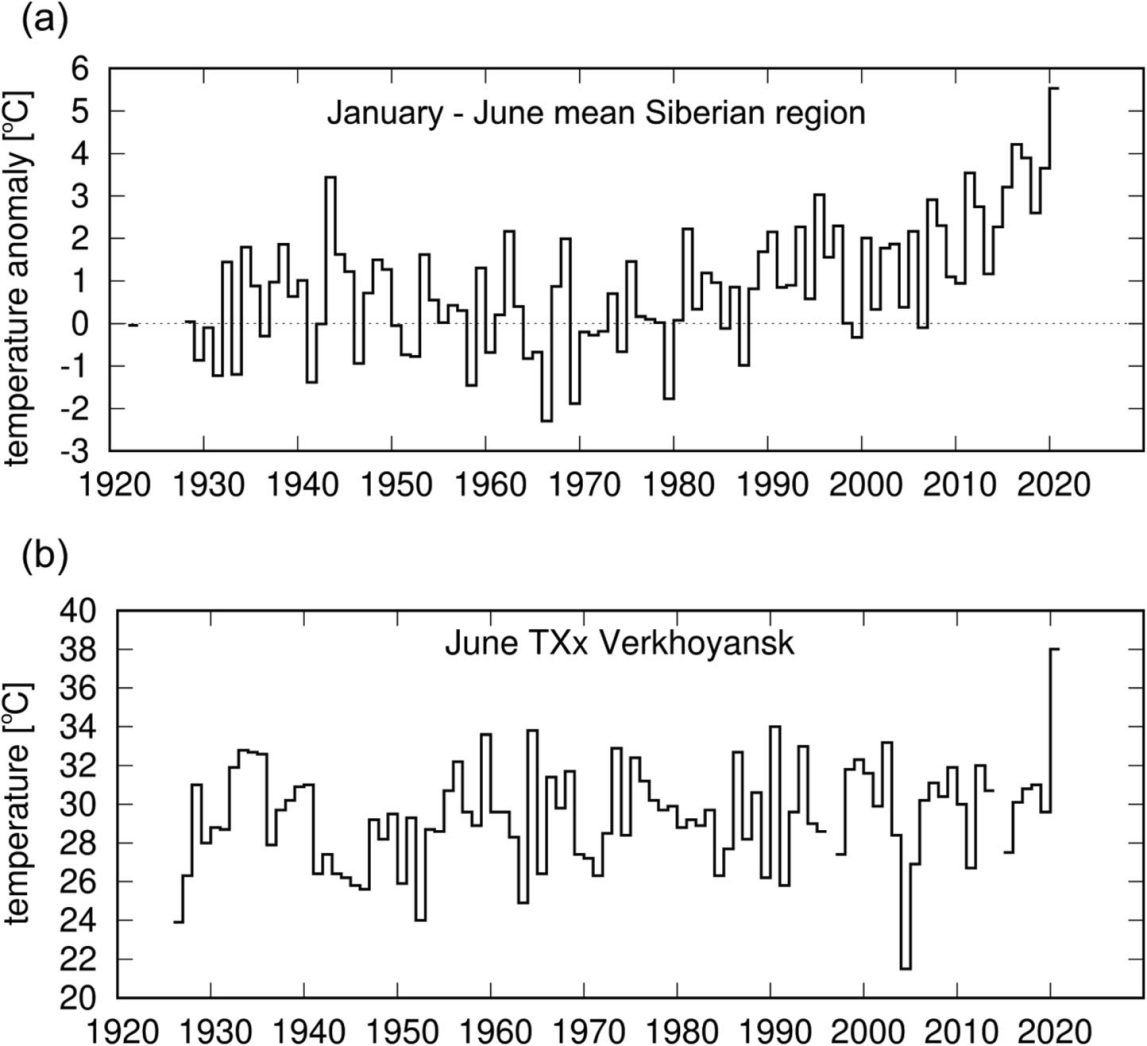
Observed time series examined in this study. 2020 values are a record in both series. **a** GISTEMP anomalies (to 1951–1980) of the near-surface air temperature [°C] for January–June 2020. **b** The observed series of maximum June daily maximum temperatures (June TXx) at Verkhoyansk

To investigate potential trends in the frequency of occurrence of prolonged Siberian high temperatures, similar to the first half of 2020, we choose to analyse January–June averaged 2-m temperature over land in the region 60°N–75°N, 60°E–180°E (region Fig. 1 (a), series Fig. 2 (a)). This region covers most of Siberia and includes the area affected by the 2020 spring monthly anomalies and Verkhoyansk, the station where the daily maximum temperature record was broken in June. The region is chosen to be representative of Siberia and, to avoid selection bias, is deliberately broader than the region that experienced the highest January–June temperatures in 2020. The January–June climatological mean temperatures are also relatively homogeneous across the study region (Fig. S3).

To investigate if human-induced climate change played a role in increasing the likelihood of the record-breaking temperature at the station Verkhoyansk, we analyse June maximum values of daily maximum 2-m temperature, i.e. the maximum temperature of the hottest June day (June TXx), at the location of the station Verkhoyansk (Fig. 2(b)). Rather than analyse summer maxima, we restrict it to the month of June because there is a strong seasonal cycle in temperature that peaks in July (Fig. S4).

Our analysis follows the approach taken by the World Weather Attribution (WWA) group (see Section 2). The aim of the study is to provide a rapid assessment of changes in frequency and intensity (temperature change at fixed probability) of the event. We approach that aim using both observational trends and a large sample of climate models with differing representations of climate processes and response to external forcing and also require that each model pass a validation step against observations. To that end, after describing the data and statistical methods in Section 2, we go on to perform initial observational analyses in Section 3. The observational analysis gives us our first estimates of frequency and intensity changes but also provides parameters that form part of the statistical validation each model must pass in Section 4. Section 5 then presents model analysis results which are combined with the observational estimates to form our synthesis results. In Section 6, we discuss our results in light of regional exposure and vulnerability to this hazard.

## Data and methods

In this section, we present details of the statistical methods and datasets to which they are applied (both observational and model output).

### Statistical methods

We first perform an analysis of time series from observational gridded data sets and station data where long records of observed data are available followed by analyses of climate model output with the same method and for the same quantities. The observational analysis plays two roles, giving us an assessment of changes that is independent of numerical climate models (but not attributed to anthropogenic climate change) as well as providing validation criteria that must be satisfied by all models included in the subsequent model-based attribution analysis. We also repeat the observational analyses with alternative datasets or methods where possible.

The WWA methodology, Philip et al. (2020a), has been used in previous publications, for example, in Van Oldenborgh et al. (2018) (heat extremes) or Van der Wiel et al. (2017) (precipitation extremes). The variable of interest is assumed to be described by a nonstationary distribution whose location (or location and scale for precipitation) changes with time and whose other parameters may be assumed constant. For regional temperature, a linear relationship with global mean surface temperature (GMST) is often well motivated (so-called pattern scaling, Tebaldi and Arblaster (2014)) and is used to describe changes in the location parameter. This fit also provides parameters whose values are used in the statistical validation of the climate models (Section 4). Observational changes in probability and intensity of events at the event threshold may then be found by evaluating the distribution for different values of the covariate series. For the early-industrial climate baseline, we take the year 1900 and for the current climate the year 2020. An equivalent analysis is then performed for each climate model passing the validation step in which each model’s own ensemble mean GMST is used as a covariate. As a means of bias correcting each model individually, we use a threshold with return level in that model corresponding to the return time of the event resulting from the observational analysis. Finally, the observational and model estimates of PR and intensity change are combined in a synthesis assessment (Section 5).

Following this method, we perform an attribution analysis for two event definitions, the January to June mean temperature over the Siberian region (land points only) and June TXx at Verkhoyansk, but also for two different periods in time: using data up to the event year, 2020, to attribute the current event and using model data up to 2050 to examine how such events are likely to evolve into the future. The PR for 2020 and 2050 are both given with respect to 1900.

Mean January to June temperatures over the Siberian region are taken to be normally distributed around a location parameter that covaries with 4-year smoothed GMST, as this quantity is averaged over both space and time. June TXx at Verkhoyansk is a block-maximum variable and so is taken to follow a generalized extreme value (GEV) which shifts with the same GMST covariate. We check residuals of the linear regression to 4-year smoothed GMST for obvious violation of the linear relationship (examination of scatter), heteroscedasticity (by 15-year running mean of standard deviation of residuals) and anomalous autocorrelation.

Confidence limits (95%) on the fit parameters, PR and intensity are produced via a 1000-member bootstrap (with a constraint that the GEV shape parameter ∣*ξ* ∣  ≲ 0.4 enforced via a Gaussian penalty in the fit).

Different groups of models are assessed using the same basic approach but with minor methodological differences (e.g. using different methods to assess uncertainty). These are described in the Supplementary Information (SI).

### Observational data

For the assessment of the large region, we use two gridded datasets: ERA5, the latest global reanalysis product from ECMWF over 1979–2020 (Hersbach et al. 2019) and GISTEMP 250-km anomalies (to 1951–1980, available regionally from 1923 onward), from the National Aeronautics and Space Administration (NASA) Goddard Institute for Space Science (GISS) surface temperature analysis with 250-km decorrelation scale (Hansen et al. 2010).

As a measure of climate change, we use the (4-year low-pass filtered) global mean surface temperature (GMST), where GMST is taken from the National Aeronautics and Space Administration (NASA) Goddard Institute for Space Science (GISS) surface temperature analysis (GISTEMP, Hansen et al. 2010).

The meteorological station Verkhoyansk (67.55°N, 133.38°E) is located in the area of the local airport at an absolute elevation of 138 m. The station was established in 1869 and provides continuous observations until now. Before 1926, the fraction of missing data is large and mainly in summer, and so maximum daily temperature observations were used starting from 1926. The fraction of June days missing data since 1926 is less than 2% and several relocations of the station since this date are not judged to have compromised the homogeneity of the record (see SI). We therefore chose to analyse June TXx from 1926 onwards (Fig. 2 (b)). We obtained the historical data via the Global Historical Climate Network Daily dataset (Menne et al. 2016) and the All-Russian Research Institute of Hydrometeorological Information—World Data Center (RIHMI-WDC) (Bulygina et al. 2014) while the data for 2020 were provided by the Russian National Hydrometeorological Centre (meteoinfo.ru) and checked with operational records from the national weather monitor (pogodaklimat.ru).

We make further remarks about the reliability of the record Verkhoyansk value in the SI.

### Model and experiment descriptions

To attribute the observed changes to anthropogenic emissions of greenhouse gases and aerosols, we use a large collection of global climate models, which includes stand-alone higher-resolution ensemble simulations run specifically for the purposes of event attribution as well as a large number of representatives of the CMIP5 and CMIP6 collections. For each model considered, only those passing the validation steps contribute results to the corresponding analysis. More details on the stand-alone and model collections are available in the SI.

EC-Earth (Hazeleger et al. 2012) is a coupled atmosphere-ocean model with a resolution of T159 (about 125 km). It is a 16-member ensemble of continuous simulations from 1860 to 2100 and is used as per the CMIP5 historical setup until 2005 and as per the RCP8.5 scenario from 2006. The MPI-ESM1–2-HR earth-system model was developed by the Max Planck Institute for Meteorology (Mauritsen et al. 2019; Mueller et al. 2018). It is a coupled global climate model. Here an ensemble of 10 CMIP6 realizations in the HR resolution (atmosphere spectral T127, roughly 100 km grid size, on 95 vertical levels) is analysed, using the SSP3-7.0 scenario (O’Neill et al. 2016) for the period 2015–2100. HadGEM3-A is the Hadley Center Atmosphere and JULES land model with prescribed sea surface temperatures and sea ice concentrations (Ciavarella et al. 2018). Horizontal resolution N216 is ~60 km mid-latitudes with 85 vertical levels including a resolved stratosphere. MPI-GE is the Max Planck Institute for Meteorology Grand Ensemble, an ensemble of 100 realizations of the Max Planck Institute Earth System Model in the low-resolution set-up (atmosphere spectral T63, roughly 210 km grid size), run with varying initial conditions (Maher et al. 2019).

CMIP5 is the 5th generation Coupled Model Intercomparison Project (Taylor et al. 2012) representing models developed by many institutes from around the world. From the CMIP5 collection of global climate models, we consider 29 models, using the historical and RCP8.5 experiments spanning the period between 1850 and 2100. CMIP6 is the 6th and latest generation Coupled Model Intercomparison Project (Eyring et al. 2016). From CMIP6, we consider a further 38 models (contributing 200 simulations on their own) using the historical and SSP5–8.5 projection experiments, again spanning the years 1850 to 2100.

An alternate analysis was performed using 7 models with initial-condition large ensembles (SMILE) (Deser et al. 2020). These are mostly CMIP5-class models re-run with larger sample sizes. This enables us to fit distributions directly to the data at a given year as a check on the main (covariate) method used for the study. The SMILE have varying ensemble sizes (16 to 100, totalling 286 simulations of historical and RCP 8.5 simulations).

## Observational analysis

The observational analysis of the station data is associated with very large uncertainties for the PR rendering the interpretation of the attribution results difficult. Confidence is much higher in the assessment for the regional event where both data sets used give very similar results. The results of the assessment of changes in event PR and intensity are presented in Table 1. Very large values that can be encountered for return times (larger than the length of the data set) and also for PR (which may be effectively unbounded above) are stated only as an indication, and where possible, we make the more conservative statement that the value is likely to be greater than the lower bound of our results. The consistency of two quantities will be understood to refer to a statement about the overlap of their 95% confidence intervals.

**Table 1:** Results of statistical analysis of observations for the Siberian region (ERA5 reanalysis and GISS observational anomalies) and the Verkhoyansk station data (two methods), comparing 2020 with 1900. Return periods for 2020 observed events are obtained from fits using dates in the first column used to set individual model bias-corrected event thresholds (via the corresponding return level) in the model analyses to follow. The numbers in parentheses indicate the 95% confidence interval upper and lower bound, respectively, except where only lower bounds are given

Dataset	Event magnitude	Return period [years]	Probability ratio PR	Change in intensity *ΔI* [°C]
Siberian region January–June mean temperature ERA5 (fit to 1979–2019)	−8.759 °C	130 (29, 2800)	>2.3 × 10^4^	4.08 (2.4, 5.6)
Siberian region January–June mean temperature GISS 250 km anomalies (fit to 1916–2019)	+5.525 °C	190 (48, 1840)	> 3600	2.85 (2.1, 3.5)
Verkhoyansk June TXx (fit to 1926–2019)	38 °C	> 140	> 2.8	1.04 (0.35, 3.4)
Verkhoyansk June TXx (fit to 1926–2020) (MF method)	38 °C	884 (>115)	> 7.3	1.63 (1.0, 2.3)

**Fig. 3:**
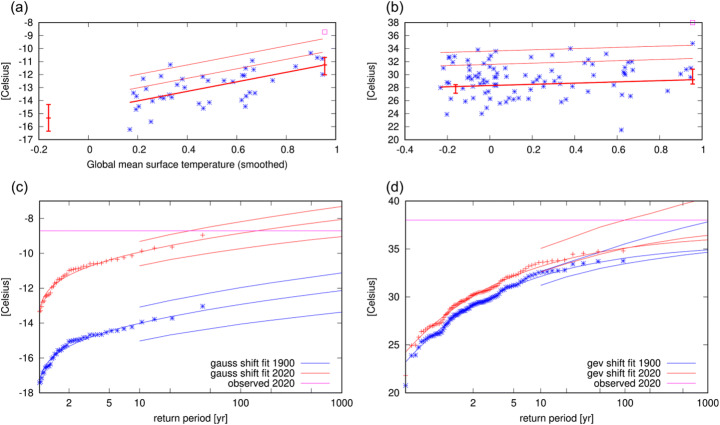
Upper panels: fit results of observed temperatures against 4-year smoothed GMST observations for **a** ERA5 Siberian region mean January–June temperatures and **b** Verkhoyansk max June daily maxima. Observational data points (blue stars), fitted location parameter (thick red line), the 6- and 40-year return values (thin red lines) and the 2020 event (magenta square). Vertical bars indicate the 95% confidence interval for the position parameter at the two reference years 2020 and 1900. Lower panels: return level versus return times for **c** ERA5 Siberian region mean January–June temperatures and **d** Verkhoyansk max June daily maxima. The data is plotted twice, being shifted with smoothed global mean temperature up to 2020 (red data points and fit with confidence interval) and down to 1900 (blue data points and fit with confidence interval), with the magnitude of the 2020 event shown as a horizontal magenta line

For the regional analysis, the covariate approach was applied first to the ERA5 reanalysis (beginning 1979, as per the methods above) with a Gaussian parametric fit returning a scale parameter (standard deviation, Table S1) of 1.0 (0.8, 1.2) °C and return period for the Siberian region January–June 2020 mean of 130 (30, 2800) years, as shown in Table 1 where numbers in brackets give bounds of the 95% confidence interval. Also in Table 1 are the event magnitude in the ERA5 dataset and results for the PR of the 2020 event and associated shift of the distribution. Figure 3 depicts the regression (a) against smoothed GMST and return levels versus return time plot (c) of this fit. The figure shows that the Gaussian distribution describes the data well. We find a very large PR value that requires a huge extrapolation of the 40 years of data, with even a lower bound of formally PR > 2×10^4^. The change in intensity since 1900 (extrapolated) is 4.1 (2.4, 5.6) °C. For the return period of the 2020 event, the best estimate and both bounds are borderline well defined at three times the length of the series, and so we choose the best estimate of 130 years as the threshold with which to define the large scale event for the model analysis.

We compared the reanalysis product with the same method applied to the longer GISS 250 km anomaly dataset (anomalies to 1951–1980) beginning in 1916. The value of the scale parameter (which is also found in Table S1) is very similar to that of the ERA5 analysis, and the return time 190 (50, 2000) years (Table 1) is also consistent between the two datasets. The GISTEMP dataset indicates a similarly large PR value with PR > 3600 and with a lower albeit still consistent change in intensity of 2.9 (2.1, 3.5) °C. These values are also summarized in Table 1.

Along exactly the same lines to the regional analysis, we fitted the June TXx values to a GEV of which the location parameter varies linearly with smoothed GMST series (Fig. 3 (b), parameters Table S2). To fit a GEV distribution to the station data, we apply two slightly different statistical methods to the same data to investigate the sensitivity of the result to the fit. The first and primary method is the WWA method implemented on the KNMI Climate Explorer (climexp.knmi.nl) (fit shown Fig. 3 (d)). The second method variant, due to Meteo-France (denoted MF), is conducted as a check on the same dataset (both described in the SI). Note that the MF method is Bayesian in nature and so uses the latest (2020) datapoint in the fit while all other analyses in this study are out of sample, fitting data up to 2019 only.

The threshold is the June 2020 TXx event of 38.0 °C (see Table 1). As the best estimate return time of the 2020 event in our primary method was undefined, we use the rounded value of the lower bound of 140 years to define the station event return time. The MF method has a smaller lower bound than this (115 years), together with a well-defined but large best estimate and upper bound, but appears not to be inconsistent. Changes in intensities from the two methods, which do not suffer from undefined values, are consistent at 1.0 (0.4, 3.4) °C for Climate Explorer and 1.6 (1.0, 2.3) °C for MF. The PR given by both the Climate Explorer and MF method have infinite best estimate and upper bounds (lower bounds of 3 and 7, respectively), indicating how extreme this event was in the observational record.

## Model validation

The climate models (see description in Section 2.3) were subjected to a model validation scheme containing both physical and statistical components before inclusion in the attribution analyses. Models were assessed according to criteria to determine whether they represent the event well, allowing for a constant bias correction.

To this end, the seasonal cycle of modelled temperature was compared to the observed seasonal cycle of the Siberian region and Verkhoyansk station. For Verkhoyansk, this is particularly important as climatological peak daily temperatures occur in July, not June. The spatial pattern of observed and modelled climatology was compared to check for gross errors. Regional model trends are also compared with the sign of the observed trend. Lastly, the fit parameters of the statistical distribution were compared with the observed fit parameters. According to these criteria (detailed further in the SI), each model may be judged ‘good’, ‘reasonable’ or ‘bad’. If enough models are judged ‘good’ then the analysis is restricted to ‘good’ models, or else ‘reasonable’ models may also be considered.

For the Siberian region, 50 out of 71 models achieved a ‘good’ evaluation (Table S1), which was judged a sufficient sample on which to conduct the attribution analysis.

For Verkhoyansk station, 33 out of 55 considered models were used (Table S2), being those which were evaluated to be ‘good’ (the best estimate of the fit parameter for the models is within the confidence interval of the observed parameter estimate) or ‘reasonable’ (the confidence intervals of the model and observed parameters estimates overlap, but the best estimate of the models is outside the 95% confidence interval in the observations). One further model (MIROC-ES2L) was excluded for possessing a trend that was both inconsistent with the observed trend and also considered to be unphysical. We chose to include also the ‘reasonable’ models because only 7 models from a single category (CMIP5) evaluated as ‘good’, which would have provided too little exploration of model uncertainty.

## Multi-method multi-model attribution and synthesis results

In this section, we discuss the model-based PR and intensity changes and synthesize them with the observation-based results already obtained in Section 3.

Model-specific bias-corrected temperature thresholds used to calculate PR are decided as per Section 2.1 using observed return times found in Table 1. For the Siberian region, the ERA5 analysis, which was available for analysis immediately after the event, was used to determine the return time, for which we took the rounded best estimate value of 130 years. For Verkhoyansk, the analysis from the Climate Explorer method was used to determine the return time. Here the best estimate was indeterminate and so the well-defined rounded lower bound of 140 years was used.

For both event definitions, we calculated the PR and intensity values of the event in the observations and the models, using only models that passed the evaluation (Section 4). Model results were synthesized with one another and then with the combined observational results to give an overarching attribution statement, following the same methodology as in Philip et al. (2020a, 2020b) and summarized briefly here. The synthesis confidence limits represent contributions arising in the model results from two sources: finite sampling of ‘weather noise’ within each model and from biases between models, which is here called ‘model spread’ (part of the model uncertainty).

**Fig. 4:**
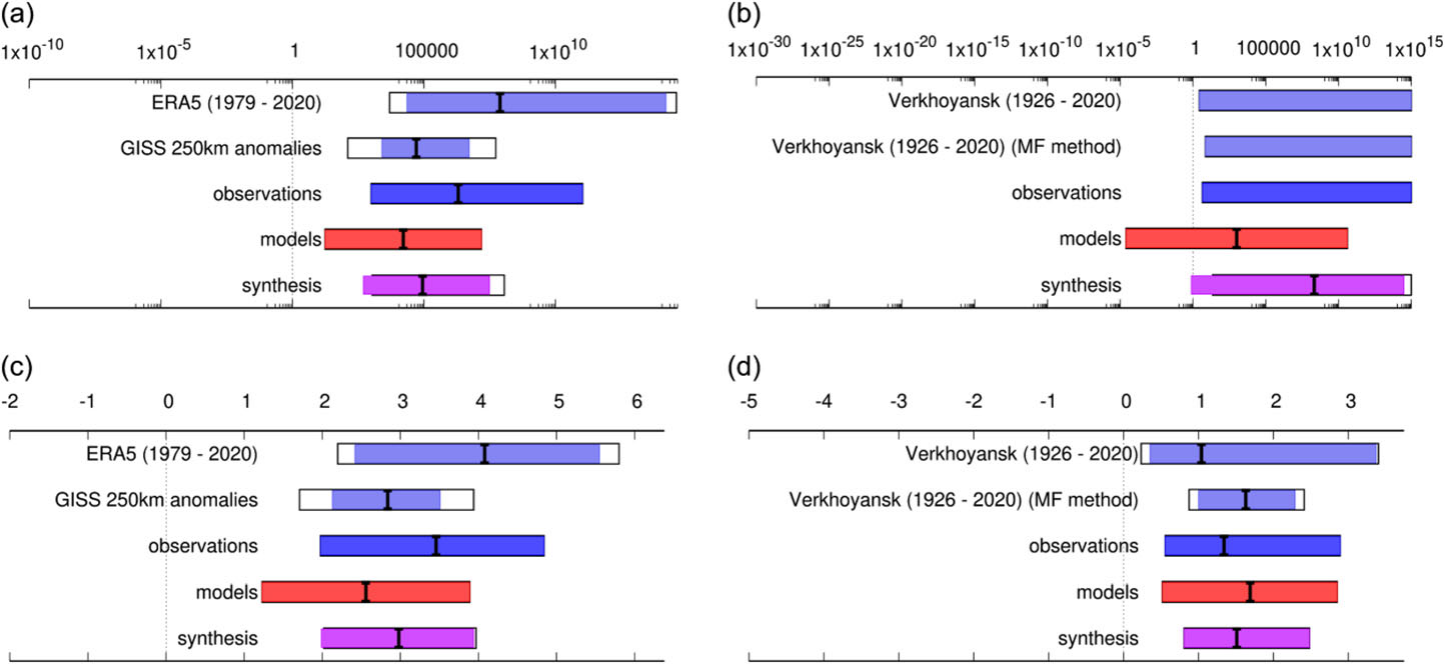
Synthesis of probability ratios (PR) ((**a**), (**b**)) and changes in intensity in °C ((**c**), (**d**)) from the attribution analysis of January–June mean Siberian region temperatures (left) and June TXx at Verkhoyansk station (right). For interpretation of filled/unfilled 95% confidence intervals, see main text. For results including all models, see SI (Figs. S5, S6 and Tables S3, S4). Date ranges indicate full dataset length available for analysis

First, the observations were combined by averaging the best estimate, lower and upper bounds, as the natural variability is strongly correlated (both are based on the same observations over 1979–2020). The difference is added as representation uncertainty (white extensions on light blue bars, Fig. 4).

Second, the models were combined by computing a weighted average (using inverse model total variances), as the natural variability in the models, in contrast to the observations, is uncorrelated. However, due to the model spread being larger than expected from variability due to sampling of weather noise alone, a model spread term was added to each model in addition to the weighted average (white extensions on the light red bars, Figs. S5, S6), to account for systematic model errors. For the PR in the Verkhoyansk analysis, we can run into the problem that the 2020 event is often above the upper bound of the probability distribution in 1900. The upper bound is due to the negative shape parameter of the GEV distributions (that always occur for daily heat wave analyses). The value for 2020 being above this upper bound formally means that the event would have been impossible in that climate. This is indicated by ‘inf’ values for the PR (Table S4). The results including this value are not mathematically well defined and so are intended merely to indicate the possibility that the event was physically impossible in a 1900 climate. However, this does depend on the assumptions made in the analysis.

Third, the synthesized results of observations and models are consistent and are therefore combined into a single result in two ways: (i) the model uncertainties beyond the model spread were neglected and the weighted average of models and observations computed (magenta bar, Fig. 4); (ii) as model bias can be larger than model sampling uncertainties, the more conservative estimate of an unweighted average of observations and models was computed (white box around the magenta bar, Fig. 4).

### Event in the climate of 2020

The synthesis of 50 ‘good’ models combined with observations shows with confidence that PR for the 6-month prolonged heat in Siberia is large (Fig. 4 (a)), with a lower bound of almost 500 and best estimate around 90,000, making the event virtually impossible in a 1900 climate. All ‘good’ models had lower bounds of the PR estimates well above 1, i.e. there is an agreement that the event probability has increased due to rising regional temperatures. Furthermore, there is a large degree of consistency between observational and model analyses for both PR and changes in intensity. Therefore, we are confident of the overall result. The average of the best estimates of the two observations has a larger shift in intensity (around 3.5 °C) than the model average best estimate (around 2.5 °C), although the longer observational dataset (GISTEMP; 2.9 °C) is closer to the models, and the two observational values are well within each other’s uncertainty estimates (Fig. 4 (c)), so there is no evidence of a recent acceleration of the trend beyond natural variability.

An event like the extreme temperature of 20 June 2020 at Verkhoyansk station has synthesis best estimate PR of 200 million, while still including the possibility of PR < 1 (Table 3, Fig. 4 (b)). The precise value (or even the order of magnitude) of this best estimate is not to be taken seriously given that it incorporates diverging values. Additionally, there is less agreement between models and observations, the latter having an infinite best estimate and upper bound PR and lower bound of 4.5.

**Table 2:** Same as for Table 2 but for the analysis of June TXx at Verkhoyansk

Dataset	Probability ratio PR [−]			Change in intensity [°C]		
	Best estimate	Uncertainty: weighted average	Uncertainty: unweighted average	Best estimate	Uncertainty: weighted average	Uncertainty: unweighted average
Verkhoyansk (fit to 1926–2019)	∞	>2.8	>2.8	1.04	(0.35, 3.4)	(0.24, 3.4)
Verkhoyansk (fit to 1926–2020) (MF method)	∞	>7.3	>7.3	1.63	(1.0, 2.3)	(0.88, 2.4)
Observation average	∞	>4.5	>4.5	1.34	(0.56, 2.9)	(0.56, 2.9)
Model average	1.02E+03	>3.2E-05	>3.2E-05	1.69	(0.52, 2.9)	(0.52, 2.9)
Synthesis	2.05E+08	>0.94	>23	1.51	(0.81, 2.5)	(0.81, 2.5)

**Table 3:** Synthesis of probability ratios (PR) and changes in intensity from the attribution analysis of January–June mean temperature in the Siberian region, comparing the 2020 event with 1900 climate. The weighted average uncertainty range corresponds to the magenta bar and the unweighted average uncertainty range to the white box, of the synthesis bar in Fig. 4

Dataset	Probability ratio PR [−]			Change in intensity [°C]		
	Best estimate	Uncertainty: weighted average	Uncertainty: unweighted average	Best estimate	Uncertainty: weighted average	Uncertainty: unweighted average
ERA5	7.73E+07	>2.3E+04	>5.9E+03	4.08	(2.4, 5.6)	(2.2, 5.8)
GISS 250-km anomalies	5.11E+04	>2.5E+03	>130	2.84	(2.1, 3.5)	(1.7, 3.9)
Observations average	2.52E+06	>1.5E+03	>1.5E+03	3.46	(2.0, 4.9)	(2.0, 4.9)
Model average	1.63E+04	>18	>18	2.56	(1.2, 3.9)	(1.2, 3.9)
Synthesis	8.87E+04	>500	>1.10E+03	2.98	(2.0, 3.9)	(2.0, 4.0)

These differences are due to the very large uncertainties involved, illustrated by the very wide observational and model 95% confidence intervals. The observational analysis is based on a smaller dataset (a series of length O(100)) than the models (ensembles), and PR estimates will be subject to greater sampling uncertainty, particularly the upper bound of the bootstrapped fitting procedure. This issue, explored recently by Paciorek et al. (2018), provides evidence that our observational confidence intervals for PR may be overestimated. Nevertheless, we do not see a practical alternative means of estimating the confidence intervals in this method, which is based on historical series and transient simulations with potentially strongly correlated estimates of past and present probability distributions.

While the Verkhoyansk PR results are of low confidence, encompassing the possibility of ‘no change’, the synthesized results for the change in intensity do confidently show a positive anthropogenic shift in temperature. The model synthesis intensity change 1.7 °C (0.5–2.9 °C) is consistent with the intensity change from observations of 1.3 °C (0.6–3.0 °C), and the model + observed synthesis value is 1.5 °C (0.8–2.5 °C). We can also see from Fig. S6 and Table S4 that it is mainly the added, conservative model spread component of uncertainty that takes model PR < 1 in most cases and that most individual models in fact confidently give PR > 1. Considering both this and the intensity results therefore increases the confidence that the true value of the PR is indeed above 1.

The weighting used to add model uncertainty in the synthesis makes it possible that models with very large PR are down-weighted unnecessarily by the synthesis algorithm, which assumes log-normal distributions, so that we could expect larger values to be closer to the truth. Removing the weighting for model uncertainty gives a PR > 4600 (using all good models) so that the good models on their own are confident of a very large PR value for June TXx at Verkhoyansk. We conclude that the presence of the upper bound in the station analysis exposes weaknesses in our synthesis methods that make the results even more uncertain than the algorithm itself indicates.

A parallel analysis of 7 SMILE large ensembles was also conducted using a different method to the covariate approach (see SI). Instead, the large ensemble values at 2018–2022 could be used directly in the parametric fit and compared with the same from an earlier epoch (1950–1954). Due to the shorter experiment length, the epoch in the 1950s was chosen as a baseline climate in place of the 1900 baseline used for the rest of the analysis.

The SMILE results, synthesized over the 7 models with the same method above, give a Siberian region PR (2020 to the 1950 baseline) with best estimate (95% confidence limits) of 2000 (700–10,000). For Verkhoyansk, the analysis gives 3.3 (>1.4) although 5 out of the 6 models with results have lower bounds less than unity. For complete results for the individual SMILE models, see Tables S5, S6 and Fig. S7.

These values for both the Siberian region and Verkhoyansk would be even larger still if the analysis could be conducted to the same 1900 baseline as the models using the covariate approach. Using this different method of analysis, we would draw very similar conclusions regarding the change in the likelihood of the regional Siberian heat, and so this provides semi-independent evidence for the near impossibility of this event in the natural world.

### Event in the climate of 2050

The best estimate synthesis PR increases by year 2050, compared to 2020, by another 3 to 4 orders of magnitude, although the lower bound of the PR is less than that for 2020 (see Fig. S8). Given that the probabilities of occurrence in the climate of 1900 are extremely small, the uncertainties in PR are so large (due to division by almost zero) that the precise figures are not well defined. What is clear is that the PR will have increased further in 2050. The synthesis change of intensity for 2050 is 4.9 °C (2.4 to 7.3 °C) which is an increase in best estimates of around 1.9 °C over the next 30 years. In other words, a hot spell with a 140-year return time in 2050 would be expected to be about 2 °C warmer than today.

The PR values for 2050 are an order of magnitude or more larger again, at 60,000 (20,000–300,000), repeating the picture of a dramatically increasing probability of occurrence with projected regional temperature rises.

## Discussion

A large, rapid multi-method attribution study, supported by observational and large ensemble model analyses, indicates with high confidence that extremely warm periods such as the 6 months of January–June 2020 over the Siberian region would have been at least 2 °C cooler in a world without human influence. Similar events have a best estimate return time in the current climate of around 130 years and are now more than 500 times as likely to occur as they would have been at the beginning of the twentieth century, with the best estimate orders of magnitude larger. By 2050, we expect such a regional warm period in the first 6 months of the year to be at least another 0.5 °C warmer and possibly up to 5 °C warmer, with similar 6-month regional temperatures becoming correspondingly more frequent.

Even granted the frequency with which event attribution studies are conducted on heat events, it is difficult to find a similarly strong result for the PR of a regional event in the literature. Of the studies related to heat previously conducted by the WWA group, none have lower bounds on PR of even the same order of magnitude. This high signal to noise of temperature increases and reflects both the size and time scale of the region averaged over but also the magnitude of the climate change itself.

Statements regarding PR of the very high June daily maximum temperatures (38 °C) which were reported at Verkhoyansk can be made only with lower confidence. The bounded nature of the distribution of extreme heat variables can be a cause of both dramatic increases in the probability of such events but also likely responsible for a large part of the associated uncertainty, partly due to statistical assumptions no longer holding. Nevertheless, results also indicate a large increase in the likelihood of such temperatures and, with more confidence, an increase in extreme daily maxima of more than 1 °C when comparing the climate of 1900 to the present day.

Slightly different scenarios (RCP8.5 vs SSP scenarios: see Model descriptions in SI) were used when modelling the future climate (see Section 2.3); however, an analysis of the partition between model and scenario uncertainty in global mean surface temperatures at 2050 in the CMIP6 collection of coupled models indicates that scenario uncertainty still plays a small role compared to model uncertainty, responsible for perhaps 35% of the spread in GMST (see Fig. 1 of Lehner et al. 2020). It has also recently been suggested that the RCP8.5 scenario will remain a close match to emissions under currently stated policies out to mid-century (Schwalm et al. 2020).

While intense heatwaves are amongst the deadliest natural disasters facing humanity today (e.g., Harrington and Otto 2020), prolonged above-average heat episodes can induce long-term environmental changes. The frequency and intensity of such heat episodes are on the rise globally, although the situation in Siberia is complicated. This a region warming much faster than the global average but mainly in winter. However, the northern study area also warms in summer. We also show that during hot days, one station also increases in temperature, although less than the half-year Siberian average that includes winter.

Geographic Siberia is a sparsely populated but vast region, home to a population of more than 33 million people who mostly live in the south but there are also some small settlements north of the Arctic circle, such as the town Verkhoyansk (population: slightly over 1100) featured in this study. Economic activities around Verkhoyansk are mostly hunting and forestry. However, the area impacted by the heat also extends northward (Fig. 1(a), Fig. S2) to the Arctic coast where the other economic sectors are developed and permafrost abounds.

Hot days in Siberia are not uncommon; however, the population is not used to extreme heat and may be more likely to suffer from common heat-induced problems such as headaches and skin conditions. Combined with other risk factors such as age, respiratory illnesses, cardiovascular disease, other pre-existing health conditions and socio-economic disadvantages, extreme heat impacts become even more acute (Kovats and Hajat 2008; McGregor et al. 2015). The results of our study confidently show significant changes in the highest daily temperatures locally that will drive an increase in the risk of these conditions in the absence of adaptation.

The Siberian environment is particularly vulnerable to prolonged above-average heat. Prolonged heat waves clearly impact the local ecosystems, resulting in, e.g. wildfires. These might expand over large areas affecting considerable loss of the resources for forestry. Moreover, wildfires emit continuous smoke which is rich in (low level) aerosols affecting air quality and also initiating important feedbacks with hot weather which can potentially enhance the temperature locally.

Increasing local temperatures also contribute to the thawing of permafrost covering most of eastern Siberia. Increasing soil temperature and active layer thickness have been observed and have accelerated over the last 15 years with summer warming trends of soil temperature being up to 0.1 °C yr^−1^ and increases in active layer depth being up to 3 cm yr^−1^ (Streletskiy et al. 2015). Loss of permafrost will only be accelerated during sustained heat events such as 2020, having critical consequences for local businesses, the well-being of local inhabitants and pollution. Streletskiy et al. (2019) project that under RCP8.5 scenario climatic changes will affect about 20% of structures and infrastructure assets, costing 16.7 bn USD and 67.7 bn USD, respectively, to mitigate.

The Russian Federation has recently published a national climate adaptation plan, targeting projects on permafrost. Moreover, a large-scale international program NEFI (Northern Eurasia Future Initiative) (Groisman et al. 2017) is focussed on mitigation in Northern Eurasian ecosystems, including permafrost, water quality, and wildfire impacts. As this study has shown, regional temperatures are projected to continue to increase over the coming three decades (at least) with associated dramatic increases in the frequency of prolonged heat events whose impacts are already being felt today.

We have demonstrated the role of anthropogenically driven temperature changes in the recent period of prolonged Siberian heat that was exceptional at both the local and wider regional scales, bringing record-breaking 6-month and daily maximum temperatures that were virtually impossible in the pre-industrial climate. Furthermore, the natural environment and human infrastructure of the region is almost uniquely vulnerable to warming and is the source of hazards to local populations as well as to the global environment. The demonstrable anthropogenic role in these events presents us with a stark warning of the impacts of human influence on climate that are hard to ignore.

## Supplementary Information

ESM 1 (DOCX 1628 kb)

## Data Availability

Time series of model and observational data used in this study are available at the following url: https://climexp.knmi.nl/siberia2020_timeseries.cgi or may be computed directly from the Climate Explorer itself or from publicly accessible data available from ESGF.
