# Supplementary material for: Prolonged Siberian heat of 2020 almost impossible without human influence
Source: Clim Change. 2021 May 6;166(1-2):9. doi: 10.1007/s10584-021-03052-w (PMC8550097; doi:10.1007/s10584-021-03052-w)
Supplement: Supplementary file 1 — (DOCX 1628 kb) [file 10584_2021_3052_MOESM1_ESM.docx]

Supplementary Information to “Prolonged Siberian heat of 2020 almost impossible without human influence”

Andrew Ciavarella^1^, Daniel Cotterill^1^, Peter Stott^1^, Sarah Kew^2^, Sjoukje Philip^2^, Geert Jan van Oldenborgh^2^, Amalie Skålevåg^3^, Philip Lorenz^3^, Yoann Robin^4^, Friederike Otto^5^, Mathias Hauser^6^, Sonia I. Seneviratne^6^, Flavio Lehner^6^, Olga Zolina^7^

^1^ Met Office Hadley Centre, FitzRoy Road, Exeter, EX1 3PB, UK

^2^ Royal Netherlands Meteorological Institute (KNMI), De Bilt, The Netherlands

^3^ Deutscher Wetterdienst (DWD), Climate and Environment Consultancy, Regional Climate Office Potsdam, Güterfelder Damm 87-91, 14532 Stahnsdorf, Germany

^4^ Météo France

^5^ University of Oxford

^6^ Institute for Atmospheric and Climate Science, ETH Zurich

^7^ IGE/UGA, Grenoble, France

^8^ P.P.Shirshov Institute of Oceanology, Moscow, Russia

**Correspondence**: Andrew Ciavarella (andrew.ciavarella@metoffice.gov.uk)

**Event definition**

In this section we present supplementary information associated with the event definition described in section 1 of the main text. First, sea level pressure anomalies are given in (Figure S1), then anomalies of snow cover in June 2020 (Figure S2), the climatology of January to June mean temperatures over Siberia (Figure S3) and the climatological seasonal cycle of monthly TXx values at Verkhoyansk station (Figure S4).

*
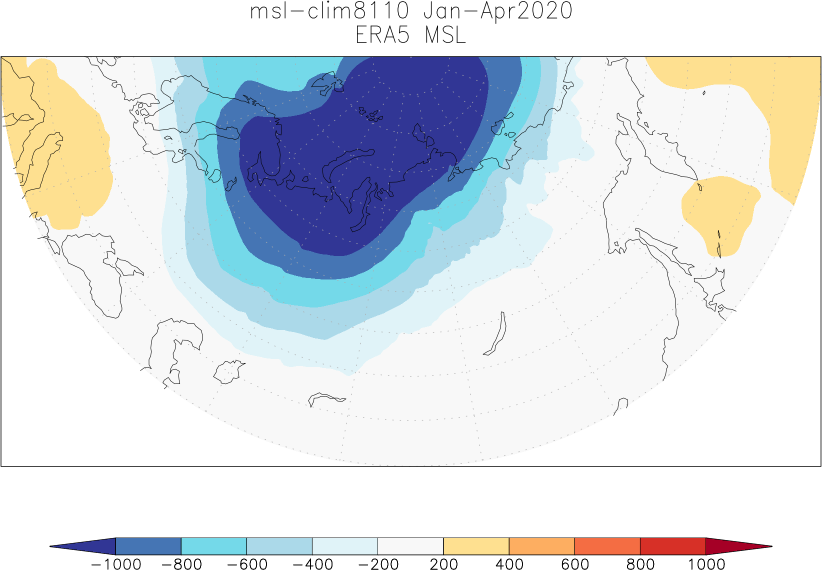

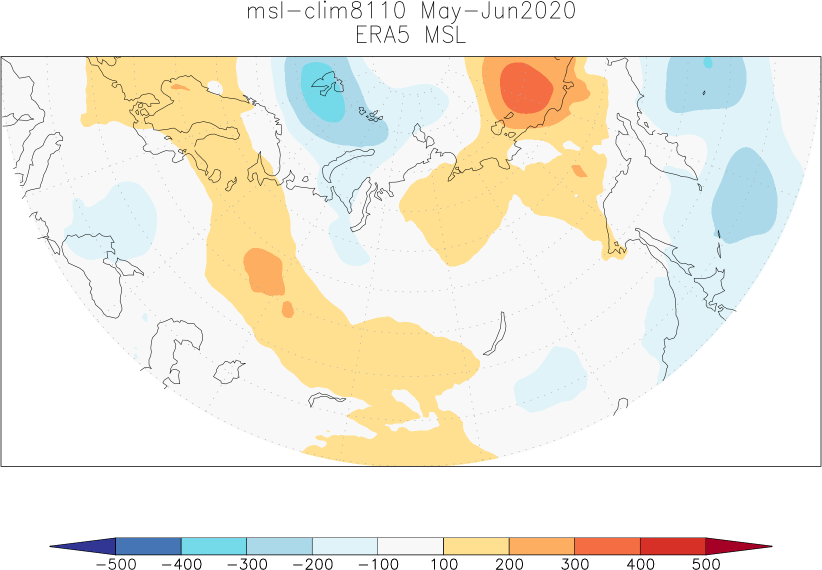
*

*Figure S1: Sea-level pressure anomalies [Pa] in January–April 2020 (left) and May-June 2020 (right). Source: ERA5.*

*
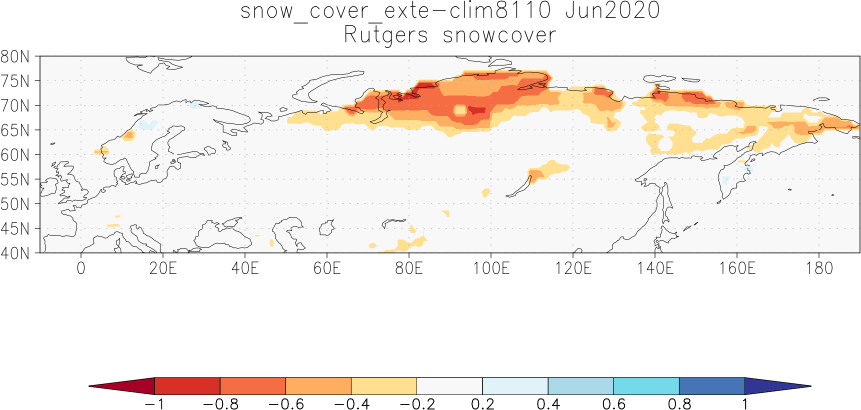
*

*Figure S2: Snow cover anomalies (w.r.t. 1981-2010) [fract.] in June 2020, the low snow cover is caused by the preceding warm months and enables much higher temperatures in June.*


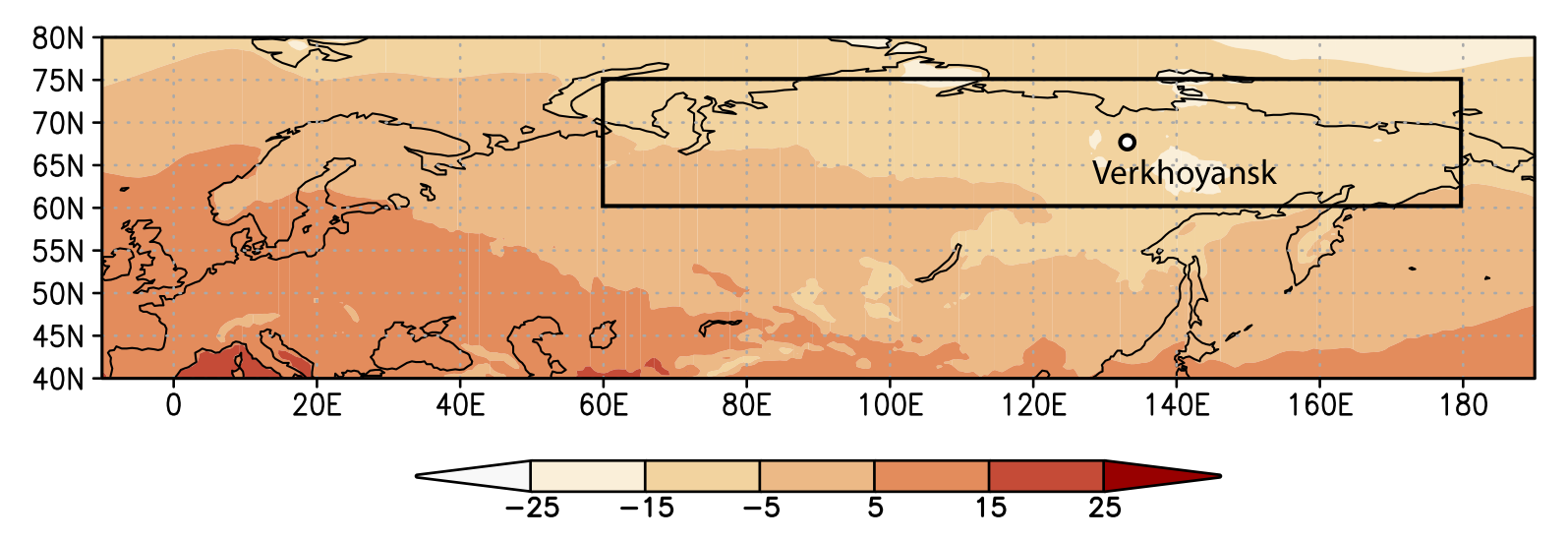


*Figure S3: ERA5 near surface (T2m) temperature [℃ ] Jan-Jun climatology for 1981-2010. The rectangle represents the study region at 60-75°N, 60-180°E. In the analysis only land points are used.*


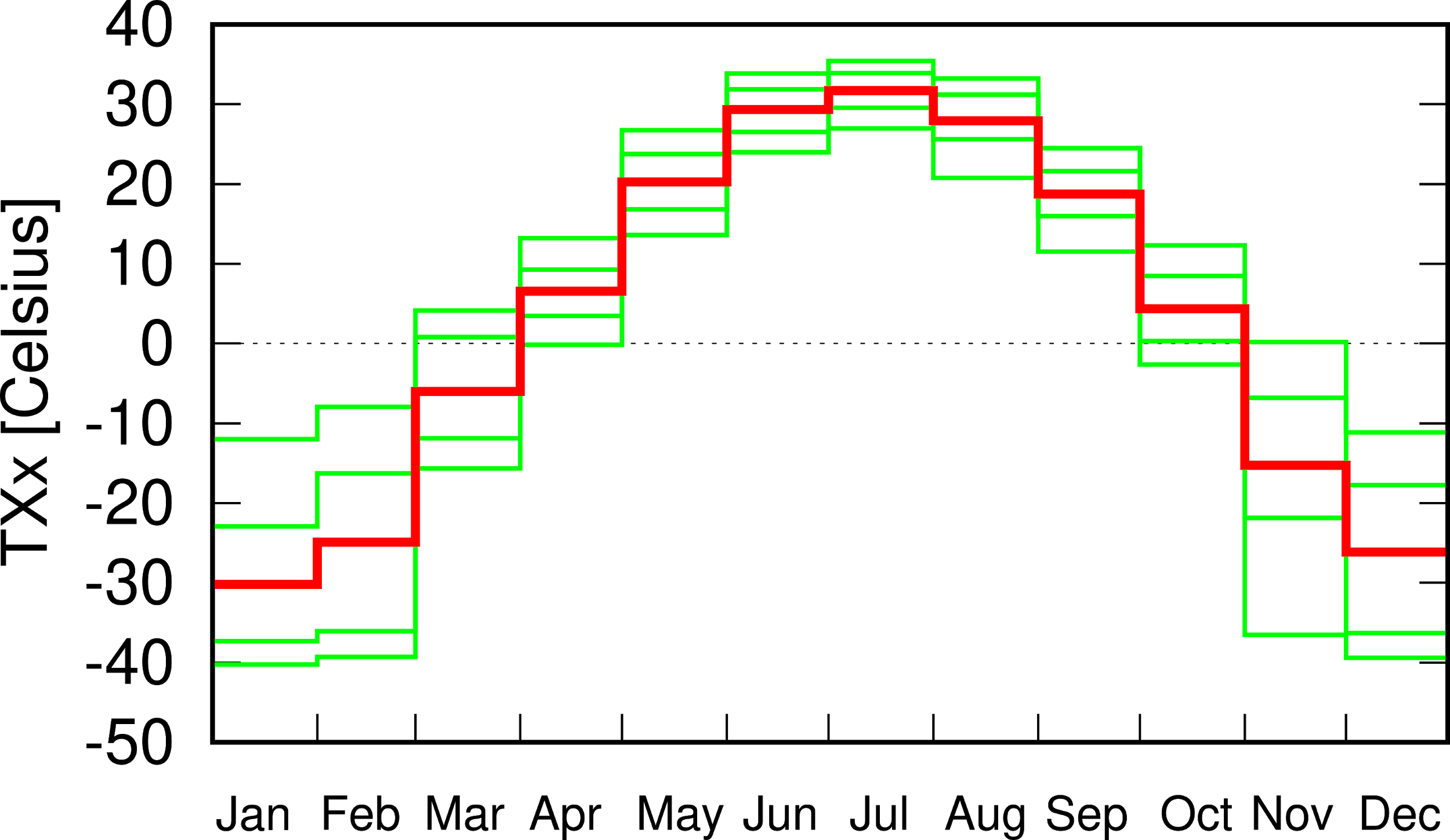


*Figure S4: The climatological seasonal cycle of monthly TXx [℃] (warmest monthly daily maximum temperature) at Verkhoyansk station. Red line is mean monthly TXx, green lines are quantiles at 2.5%, 17%, 83% and 97.5% of the distribution over the full Verkhoyansk dataset 1926 - 2020. TXx normally peaks in July, making the June record exceptional.*

**Verkhoyansk station data**

In September 1999 the station was relocated some 2.5 km southwestward from the airport. This did not result in any critical change in the station elevation (about 4 meters). Previous to this data two other major relocations were documented, specifically in October 1940 (by 1.5 km northward from its original location) and in April 1947 (by about 2.5 km southward to the airport area). Overall, the station should be considered as properly exposed, not influenced by any large infrastructure and providing a homogenous temperature record.

We can make a few further remarks regarding the reliability of the record 38°C itself on June 20^th^ , later confirmed by the Russian Meteorological agency, Roshydromet.

As we note in our article, while the ERA reanalysis does not reproduce the extreme local value, as expected, closest grid cells do possess their own record value in the vicinity of Verkhoyansk that peak over June 20^th^ to 21^st^, supporting the presence of a record extreme value and a peak of local heat. As discussed in the main text, the meteorology would also appear to support extreme temperatures and the record on the 20^th^ occurs within a period of building heat that reaches in excess of 34°C at Verkhoyansk on surrounding days, including 34.5°C on the 22^nd^, after conditions suggest peak heat has occurred.

There are a handful of stations on GHCN-D database with Tmax data on June 20^th^ that are in the wider region, but station density is low and even the closest such station is over 370km away: Ljubljana, 70.77N, 136.22E. Examining ERA5 Tmax fields over June 18^th^ – 22^nd^ we see that the spatial extent of peak heat in the reanalysis remains well within the approximate confines of the region 65-70N, 130-136E in which we find no other stations except Verkhoyansk on GHNC-D containing Tmax data. Ljubljana station, at the edge of this region, does see its peak heat on June 20^th^ at 32.4°C when Verkhoyansk has already attained 34°C on the preceding day. In fact ERA5 fields themselves suggest a peak over June 20th – 21st that could be a several degrees above temperatures either side of this, when Verkhoyansk station has exceeded 34°C. ERA5 fields also suggest that Ljubljana station would be the warmest of the other stations neighboring Verkhoyansk on June 20^th^ and we do indeed find that these others, at further distance still, have slightly lower maximum temperatures.

So although we do not have neighboring station data to directly support the very high absolute value of 38°C we do see supporting evidence that the immediate region of Verkhoyansk may well have seen peak temperatures above that of the nearest stations with data, and possibly several degrees above 34.5°C.

Finally we note that apart from defining the magnitude of the threshold we examine no other part of the analysis (apart from the Bayesian approach check of the observational analysis) is affected by the value of the event itself.

**Model descriptions**

*EC-Earth*

EC-Earth (Hazeleger et al., 2012) is a coupled atmosphere-ocean model with a resolution of T159 (about 125 km). It is a 16-member ensemble of continuous simulations from 1860-2100 and is used as per the CMIP5 historical setup until 2005 and as per the RCP8.5 scenario from 2006.

*MPI-ESM1.2-HR*

The MPI-ESM1.2-HR earth system model was developed by the Max Planck Institute for Meteorology (Mauritsen et al., 2019, Mueller et al., 2018). It is a coupled global climate model. Here an ensemble of 10 CMIP6 realizations in the HR resolution (atmosphere spectral T127, roughly 100km grid size, on 95 vertical levels) is analysed. The historical experiment is available on ESGF for 1850-2014. For the period 2015-2100 the SSP3-7.0 scenario was used. Based on the model output variable Tx (daily maximum temperature), the June TXx was computed, in addition to the average January-June temperature for the Siberian region based on the model output variable daily mean temperature.

*HadGEM3-A*

Hadley Center Atmosphere and JULES land model with prescribed sea surface temperatures and sea ice concentrations. Horizontal resolution N216 is ~60km mid-latitudes with 85 vertical levels including resolved stratosphere. Data available from 1960 onwards with 15 ensemble members 1960 - 2013 and larger ensembles thereafter for both historical and historicalNat attribution experiments. (Ciavarella et al., 2018). The historical experiment uses RCP4.5 climate forcings after 2005.

*CMIP5*

CMIP5 is a set of global climate models, developed by several institutes around the world (Taylor et al., 2012). Here a subset of CMIP5 models passing the validation steps are used, with historical and RCP8.5 experiments together spanning the period between 1850 and 2100. The list of models used is given in the validation tables section.

*CMIP6*

Data from the 6th Coupled Model Intercomparison Project (CMIP6; Eyring et al., 2016) is also assessed. Therefore, we combine the historical simulations (1850 to 2015) with the shared socioeconomic pathways (SSPs) projections (O’Neill et al., 2016) for the years 2016 to 2100. Here, we only use data from SSP5-8.5. Models are excluded if they do not provide the relevant variables, do not run from 1850 to 2100, or include duplicate time steps or missing time steps. All available ensemble members are used. For the regional analysis 38 models (200 ensemble members) are used that passed the validation tests and for Verkhoyansk 28 models (166 ensemble members).

*SMILES*

Single model initial-condition large ensembles (SMILEs) are large ensembles of mostly CMIP5-class models. While the model versions of the SMILEs are almost all included in the standard CMIP5 archive described above, the main benefit of the SMILEs is their larger sample size. This enables fitting distributions to the data at a given time step, rather than having to fit a distribution over time, which requires subtracting an estimate of the forced response. The SMILEs have varying ensemble sizes (16 to 100, totalling 286 simulations of historical and RCP 8.5) and are centrally archived in the Multi Model Large Ensemble Archive (<http://www.cesm.ucar.edu/projects/community-projects/MMLEA/>). Details and references on the archive can be found in Deser et al. (2020).

*MPI-GE*

The Max Planck Institute for Meteorology Grand Ensemble (MPI-GE) is an ensemble of 100 realisations of the Max Planck Institute Earth System Model in the low resolution set up (T63/1.9° horizontal resolution, i.e. ~210 km), run with varying initial conditions (Maher et al., 2019). Monthly data are available on the ESGF. For 1850-2004 data from the historical experiment, and for 2005-2100 from the RCP8.5 scenario were downloaded and the January-June mean temperature for the siberian region calculated.

**Methodological Variants**

### *Method of Météo-France (MF)*

The Météo-France method uses the same statistical models, but differs in two ways. MF assumes first that the covariate (here the GMST) is also random, and its confidence interval is taken into account. Secondly, the distribution of observations is fitted in a Bayesian way with a synthesis of a set of CMIP5 models as prior. The uncertainty of the covariate is inferred by the uncertainty of the decomposition of the GMST as the sum of an EBM model (natural forcings), a smoothing spline of time (anthropic forcings) and a residual Gaussian random term (natural forcings). This decomposition is applied to each model of a set of CMIP5 models, and used to fit the distribution of the variable of interest (GEV for maxima, etc). The confidence intervals are inferred by resampling 1000 times each model. Then, either the return time has already been specified and is used as a definition to calculate the PR and the ΔI for each CMIP5 model separately, or, if Météo France provides an observational analysis, the CMIP5 multi-model distribution is used as a prior to calculate the PR and the ΔI from the observations of the GMST and the variable of interest with Bayesian methods (a combination of the Gaussian conditioning theorem and the Markov Chain Monte Carlo approach, see Robin et al, 2020, in review). See also Ribes et al. (2020) and Ribes et al. (2017) for further information.

We note that while the main method does not use the 2020 value itself in the analysis the MF method does so. The philosophy of a Bayesian approach is to accept a modification (even substantial) of the statistical model on the basis of what has been observed, and to re-estimate the uncertainty on the basis of these observations. In this context, it is normal to accept the highest value, as it will update the estimate accordingly.

### *Method for CMIP6 data (ETH Zurich)*

The CMIP6 data is analysed using the same statistical models as the main method. However, the parameter uncertainty is estimated in a Bayesian setting using a Markov Chain Monte

Carlo (MCMC) sampler and not via bootstrapping. Using an affine‐transformation invariant MCMC sampler (Goodman and Weare, 2010; Foreman‐Mackey et al., 2013) we generate at least 25’000 non-independent samples. From these we calculate the best estimate of the parameters (median) and their uncertainty (using the 2.5 % and 97.5 % percentile). We use noninformative priors, except for the shape parameter of the GEV. ξ is constrained with a Gaussian prior with a standard deviation of 0.15.

### *Method for SMILES*

As a minor variant to the main method, distributions can be fitted to ensemble members of a single model initial-condition large ensemble (SMILE) at a given point in time (rather than fitting distributions across time). For a subset of CMIP5 and CMIP6 models, such SMILEs are available with ensemble members ranging from 16 to 100, depending on the climate model. This approach has the benefit that the changing background climate is taken into account automatically, such that GMST does not need to be invoked as a covariate. It also does not assume constant distributional parameters, as those change automatically as a result of the changing background climate (e.g., a forced future decrease in temperature variability associated with the loss of cryosphere at high latitudes).

All SMILE data is interpolated conservatively to a 2.5°x2.5° grid in the beginning. The SMILEs have a common start year of 1950, therefore probabilities and probability ratios (PR) are calculated for year 1950 (using pooled data from 1950-1954), 2020 (2018-2022) and 2050 (2048-2052). They are thus not directly comparable to the other results in this study, which focus on probability ratio relative to 1900 instead of 1950. Consequently, one might expect the PR of the SMILEs to be slightly smaller, as they do not include the climate change (i.e., GMST increase) between 1900 and 1950. In practice, though, the PR values are very similar given the confidence interval of the PR values.

**Validation criteria extra details**

The statistical element of the validation step involves comparison between observations and models of the parameters of the fit of the residuals from the GMST covariate. If a model’s best estimate parameter values are found to be within the 95% confidence interval (CI) of the observed values then that model is judged to be “good”. If the model best estimate is outside the observed CI but model and observed CI’s overlap then the model is judged “reasonable”. If there is no overlap of parameter CI’s the model is judged “bad”. Similar judgements are made for each of the physical validation criteria (station seasonal cycle peaks in July, plots of spatial pattern of climatology of variable judged by eye and sign of trend compared with observed trend). These are combined with the statistical criteria to form an overall judgement “good”, “reasonable” or “bad” that informs the decision on whether or not to include the model.

**Validation tables**

The full results of the validation of seasonal cycle, spatial pattern and consistency of parametric fit parameters with observed values for all model considered are presented below for the Siberian region (Table S1) and for Verkhoyansk (Table S2).

*Table S1: All models considered for analysis of Siberian region. For some models the number of ensemble members is indicated behind the model name in parentheses. Observational parameter values against which models are validated are also given at the top of the table (blue text).*

| **Model / Observations** | **Seasonal cycle** | **Spatial pattern** | **Sigma** | **Conclusion** |
| --- | --- | --- | --- | --- |
| ERA5 (fit to 1979 - 2019) |  |  | 1.036 (0.826 ... 1.197) |  |
| GISTEMP 250km anomalies (fit to 1916 - 2019) |  |  | 1.077 (0.917 ... 1.2) |  |
| MPI-ESM1.2-HR (10) | good | good | 1.094 (1.017 ... 1.167) | good |
| EC-Earth | good | reasonable | 1.131 (1.074 ... 1.179) | reasonable Gaussian fit not good near event RP |
| BCC_bcc-csm1-1-m | good | good | 0.96 (0.9 ... 1.02) | good |
| BNU_BNU-ESM | good | reasonable | 0.99 (0.91 ... 1.07) | good |
| CCCma_CanESM2 | good | reasonable | 1.3 (1.25 ... 1.35) | bad |
| CMCC_CMCC-CESM | good | reasonable | 1.02 (0.93 ... 1.11) | good |
| CMCC_CMCC-CMS | good | reasonable | 1.03 (0.94 ... 1.11) | good |
| CNRM-CERFACS_CNRM-CM5 | good | good | 1.06 (1.03 ... 1.09) | good |
| CSIRO-BOM_ACCESS1-0 | good | good | 1.15 (1.09 ... 1.22) | good |
| CSIRO-BOM_ACCESS1-3 | good | good | 1.01 (0.95 ... 1.07) | good |
| CSIRO-QCCCE_CSIRO-Mk3-6-0 | good | reasonable | 0.69 (0.67 ... 0.71) | bad |
| FIO_FIO-ESM | good | reasonable | 0.97 (0.92 ... 1.03) | good |
| INM_inmcm4 | good | reasonable | 1 (0.91 ... 1.08) | good |
| IPSL_IPSL-CM5A-LR | good | good | 0.84 (0.81 ... 0.87) | good |
| IPSL_IPSL-CM5A-MR | good | good | 0.83 (0.78 ... 0.87) | good |
| IPSL_IPSL-CM5B-LR | good | good | 0.81 (0.75 ... 0.87) | reasonable |
| MIROC_MIROC-ESM-CHEM | good | reasonable | 0.91 (0.83 ... 0.99) | good |
| MIROC_MIROC-ESM | good | reasonable | 0.97 (0.9 ... 1.03) | good |
| MIROC_MIROC5 | good | reasonable | 0.94 (0.9 ... 0.97) | good |
| MPI-M_MPI-ESM-LR | good | good | 1.1 (1.05 ... 1.15) | good, but excluded because MR-version gives same results |
| MPI-M_MPI-ESM-MR | good | good | 1.08 (1.01 ... 1.14) | good |
| MRI_MRI-CGCM3 | good | good | 1.16 (1.09 ... 1.23) | good |
| NASA-GISS_GISS-E2-H-CC | good | good | 0.9 (0.82 ... 0.96) | good |
| NASA-GISS_GISS-E2-H | good | good | 0.85 (0.81 ... 0.88) | good |
| NASA-GISS_GISS-E2-R-CC | good | good | 0.78 (0.7 ... 0.85) | reasonable |
| NASA-GISS_GISS-E2-R | good | good | 0.85 (0.82 ... 0.89) | good |
| NCAR_CCSM4 | good | good | 1.14 (1.1 ... 1.17) | good |
| NCC_NorESM1-M | good | good | 1.17 (1.1 ... 1.24) | good |
| NCC_NorESM1-ME | good | reasonable | 1.3 (1.18 ... 1.4) | reasonable |
| NSF-DOE-NCAR_CESM1-BGC | good | good | 1.19 (1.09 ... 1.28) | good |
| NSF-DOE-NCAR_CESM1-CAM5 | good | reasonable | 1.31 (1.24 ... 1.37) | bad |
| HadGEM3-A ALL | good | good | 1.01 (0.96 ... 1.05) | good |
| ACCESS-CM2 (3) | good | good | 1.33 (1.24 ... 1.43) | bad (sigma too high) |
| ACCESS-ESM1-5 (3) | good | good | 1.44 (1.34 ... 1.56) | bad (sigma too high) |
| AWI-CM-1-1-MR (1) | good | good | 1.11 (0.98 ... 1.27) | good |
| BCC-CSM2-MR (1) | good | good | 1.02 (0.9 ... 1.16) | good |
| CAMS-CSM1-0 (2) | good | good | 0.8 (0.73 ... 0.88) | reasonable (sigma too small) |
| CESM2 (5) | good | good | 1.19 (1.12 ... 1.26) | good |
| CESM2-WACCM (3) | good | good | 1.12 (1.04 ... 1.21) | good |
| CIESM (1) | good | good | 1.2 (1.06 ... 1.36) | good |
| CMCC-CM2-SR5 (1) | good | good | 1.02 (0.91 ... 1.16) | good |
| CNRM-CM6-1 (6) | good | good | 1.26 (1.19 ... 1.32) | reasonable (sigma too large) |
| CNRM-CM6-1-HR (1) | good | good | 0.99 (0.87 ... 1.13) | good |
| CNRM-ESM2-1 (5) | good | good | 1.21 (1.15 ... 1.28) | reasonable (sigma too large) |
| CanESM5 (50) | good | good | 1.19 (1.16 ... 1.21) | good |
| CanESM5-CanOE (3) | good | good | 1.17 (1.09 ... 1.26) | good |
| EC-Earth3 (7) | good | reasonable | 1.02 (0.98 ... 1.08) | good |
| EC-Earth3-Veg (4) | good | good | 0.95 (0.9 ... 1.02) | good |
| FGOALS-f3-L (1) | good | good | 1.18 (1.05 ... 1.35) | good |
| FGOALS-g3 (4) | good | good | 1.07 (1.01 ... 1.15) | good |
| FIO-ESM-2-0 (3) | good | good | 1.2 (1.12 ... 1.3) | good |
| GFDL-CM4 (1) | good | good | 0.94 (0.83 ... 1.07) | good |
| GFDL-ESM4 (1) | good | good | 0.81 (0.72 ... 0.93) | good |
| GISS-E2-1-G (1) | good | good | 1.25 (1.1 ... 1.43) | reasonable (sigma too large) |
| HadGEM3-GC31-LL (4) | good | good | 0.92 (0.86 ... 0.98) | good |
| HadGEM3-GC31-MM (3) | good | good | 0.95 (0.89 ... 1.03) | good |
| INM-CM4-8 (1) | good | good | 0.85 (0.75 ... 0.97) | good |
| INM-CM5-0 (1) | good | good | 0.81 (0.71 ... 0.92) | reasonable (sigma too small) |
| IPSL-CM6A-LR (6) | good | good | 1.05 (0.99 ... 1.1) | good |
| KACE-1-0-G (3) | good | good | 1.41 (1.31 ... 1.51) | bad (something goes wrong with Tglob) |
| MCM-UA-1-0 (1) | good | reasonable | 1.25 (1.1 ... 1.44) | reasonable (sigma too large) |
| MIROC-ES2L (1) | good | reasonable | 0.81 (0.72 ... 0.93) | reasonable (sigma too small) |
| MIROC6 (50) | good | good | 0.9 (0.88 ... 0.92) | good |
| MPI-ESM1-2-HR (2) | good | good | 1.11 (1.01 ... 1.21) | good |
| MPI-ESM1-2-LR (10) | good | good | 1.06 (1.02 ... 1.1) | good |
| MRI-ESM2-0 (2) | good | reasonable | 1.28 (1.17 ... 1.4) | reasonable (sigma too large) |
| NESM3 (2) | good | good | 1.33 (1.22 ... 1.46) | bad (sigma too high) |
| NorESM2-LM (1) | good | good | 1.34 (1.18 ... 1.54) | reasonable (sigma too high) |
| NorESM2-MM (1) | good | good | 1.17 (1.04 ... 1.34) | good |
| UKESM1-0-LL (5) | good | good | 0.99 (0.94 ... 1.05) | good |
| MPI-GE (100) | good | good | 1.08 (1.05 ... 1.1) | good |

*Table S2: All models considered for analysis of Verkhoyansk station. For some models the number of ensemble members is indicated behind the model name in parentheses. Observational parameter values against which models are validated are also given at the top of the table (blue text).*

| **Model / Observations** | **Seasonal cycle** | **Spatial pattern** | **Sigma** | **Shape parameter** | **Conclusion** |
| --- | --- | --- | --- | --- | --- |
| Verkhoyansk (fit to 1926 - 2019) |  |  | 2.51 (1.885 ... 2.586) | -0.366 (-0.31 ... -0.117) |  |
| Verkhoyansk (fit to 1926 - 2020) MF method) |  |  | 2.67 (2.33 ... 3.1) | -0.27 (-0.32 ... -0.21) |  |
| MPI-ESM1.2-HR | reasonable |  | 3.388  (3.219 ... 3.543) | -0.298  (-0.358 ... -0.27) | bad, sigma too high |
| EC-Earth | good |  | 3.012  (2.882 ... 3.131) | -0.277  (-0.305 ... -0.251) | bad, sigma too high |
| BCC_bcc-csm1-1-m | good | good | 3.24  (3.02 ... 3.44) | -0.24  (-0.3 ... -0.19) | bad |
| CCCma_CanESM2 | good | reasonable | 2.81  (2.69 ... 2.94) | -0.26  (-0.3 ... -0.23) | bad |
| CMCC_CMCC-CESM | good | reasonable | 3.13  (2.7 ... 3.47) | -0.38  (-0.45 ... -0.3) | bad |
| CMCC_CMCC-CMS | good | reasonable | 2.81  (2.55 ... 3.05) | -0.3  (-0.42 ... -0.23) | reasonable |
| CNRM-CERFACS_CNRM-CM5 | good | good | 2.39  (2.29 ... 2.47) | -0.22  (-0.25 ... -0.2) | good |
| CSIRO-BOM_ACCESS1-0 | good | good | 2.52  (2.3 ... 2.71) | -0.24  (-0.3 ... -0.19) | good |
| CSIRO-BOM_ACCESS1-3 | good | good | 2.2  (2.01 ... 2.37) | -0.28  (-0.38 ... -0.22) | good |
| CSIRO-QCCCE_CSIRO-Mk3-6-0 | good | reasonable | 2.28  (2.21 ... 2.35) | -0.24  (-0.27 ... -0.22) | good |
| INM_inmcm4 | good | reasonable | 2.95  (2.65 ... 3.24) | -0.32  (-0.4 ... -0.25) | bad |
| IPSL_IPSL-CM5A-LR | good | good | 1.85  (1.76 ... 1.94) | -0.26  (-0.29 ... -0.23) | reasonable |
| IPSL_IPSL-CM5A-MR | good | good | 1.75  (1.62 ... 1.87) | -0.22  (-0.29 ... -0.17) | bad |
| IPSL_IPSL-CM5B-LR | good | good | 1.98  (1.78 ... 2.17) | -0.31  (-0.41 ... -0.24) | good |
| MIROC_MIROC-ESM-CHEM | good | reasonable | 4.5  (3.82 ... 5.05) | -0.39  (-0.52 ... -0.32) | bad |
| MIROC_MIROC-ESM | good | reasonable | 4.58  (4.14 ... 4.99) | -0.41  (-0.55 ... -0.36) | bad |
| MIROC_MIROC5 | good | reasonable | 2.75  (2.63 ... 2.86) | -0.25  (-0.29 ... -0.22) | bad |
| MPI-M_MPI-ESM-LR | good | good | 3.19  (3 ... 3.38) | -0.25  (-0.32 ... -0.21) | bad |
| MPI-M_MPI-ESM-MR | good | good | 3.17  (2.95 ... 3.38) | -0.29  (-0.34 ... -0.25) | bad |
| MRI_MRI-CGCM3 | good | good | 2.64  (2.44 ... 2.82) | -0.23  (-0.28 ... -0.18) | reasonable |
| NASA-GISS_GISS-E2-H | good | good | 2.21  (2.01 ... 2.39) | -0.2  (-0.33 ... -0.12) | good |
| NASA-GISS_GISS-E2-R | good | good | 2.13  (1.93 ... 2.3) | -0.17  (-0.24 ... -0.1) | good |
| NCAR_CCSM4 | good | good | 3.71  (3.54 ... 3.9) | -0.32  (-0.38 ... -0.3) | bad |
| NCC_NorESM1-M | good | good | 3.6  (3.42 ... 3.8) | -0.29  (-0.4 ... -0.26) | bad |
| NSF-DOE-NCAR_CESM1-BGC | good | good | 3.49  (3.16 ... 3.81) | -0.32  (-0.46 ... -0.27) | bad |
| NSF-DOE-NCAR_CESM1-CAM5 | good | reasonable | 3.05  (2.8 ... 3.25) | -0.3  (-0.36 ... -0.25) | bad |
| HadGEM3-A | good | good | 2.67  (2.55 ... 2.81) | -0.242  (-0.32 ... -0.22) | reasonable |
| ACCESS-CM2 (2) | good | good | 2.52  (2.31 ... 2.78) | -0.3  (-0.37 ... -0.23) | good |
| ACCESS-ESM1-5 (3) | good | good | 2.17  (2.01 ... 2.35) | -0.28  (-0.35 ... -0.21) | good |
| AWI-CM-1-1-MR (1) | good | good | 3.52  (3.11 ... 4.04) | -0.28  (-0.38 ... -0.16) | reasonable (sigma too large) |
| BCC-CSM2-MR (1) | good | good | 5.48  (4.84 ... 6.2) | -0.23  (-0.31 ... -0.13) | bad? (sigma way too large) |
| CMCC-CM2-SR5 (1) | good | good | 2.5  (2.2 ... 2.87) | -0.23  (-0.34 ... -0.11) | good |
| CNRM-CM6-1 (1) | good | good | 2.21  (1.95 ... 2.56) | -0.26  (-0.36 ... -0.14) | good |
| CNRM-CM6-1-HR (1) | good | good | 2.34  (2.07 ... 2.69) | -0.25  (-0.35 ... -0.13) | good |
| CNRM-ESM2-1 (1) | good | good | 2.07  (1.83 ... 2.39) | -0.15  (-0.25 ... -0.03) | good |
| CanESM5 (50) | good | good | 2.87  (2.82 ... 2.92) | -0.32  (-0.33 ... -0.31) | reasonable (shape too small) |
| EC-Earth3 (3) | good | good | 4.02  (3.76 ... 4.33) | -0.31  (-0.34 ... -0.28) | reasonable (sigma too large) |
| EC-Earth3-Veg (4) | good | good | 3.81  (3.59 ... 4.08) | -0.33  (-0.37 ... -0.3) | bad (sigma too large; shape too small) |
| FGOALS-g3 (3) | good | good | 2.98  (2.78 ... 3.23) | -0.29  (-0.33 ... -0.23) | good |
| GFDL-CM4 (1) | good | good | 2.68  (2.35 ... 3.09) | -0.25  (-0.37 ... -0.11) | good |
| GFDL-ESM4 (1) | good | good | 2.32  (2.05 ... 2.65) | -0.37  (-0.47 ... -0.25) | reasonable (shape too small) |
| HadGEM3-GC31-LL (4) | good | good | 2.77  (2.59 ... 2.96) | -0.27  (-0.31 ... -0.22) | good |
| HadGEM3-GC31-MM (3) | good | good | 2.58  (2.39 ... 2.79) | -0.09  (-0.12 ... -0.05) | good |
| INM-CM4-8 (1) | good | good | 2.7  (2.39 ... 3.09) | -0.36  (-0.48 ... -0.24) | bad (sigma too large; shape too small) |
| INM-CM5-0 (1) | good | good | 2.78  (2.49 ... 3.16) | -0.36  (-0.45 ... -0.26) | reasonable (shape too small) |
| IPSL-CM6A-LR (6) | good | good | 2.9  (2.75 ... 3.07) | -0.25  (-0.3 ... -0.2) | good |
| KACE-1-0-G (2) | good | good | 3.72  (3.39 ... 4.13) | -0.1  (-0.18 ... -0.01) | bad (somthing wrong with the data) |
| MIROC-ES2L (1) | good | good | 2.41  (2.12 ... 2.74) | -0.25  (-0.34 ... -0.14) | good |
| MIROC6 (50) | good | good | 2.46  (2.42 ... 2.51) | -0.3  (-0.31 ... -0.29) | good |
| MPI-ESM1-2-HR (2) | good | good | 3.32  (3.03 ... 3.67) | -0.24  (-0.3 ... -0.15) | reasonable (sigma too large) |
| MPI-ESM1-2-LR (10) | good | good | 3.27  (3.14 ... 3.42) | -0.31  (-0.34 ... -0.28) | reasonable (sigma too large) |
| MRI-ESM2-0 (2) | good | good | 2.82  (2.57 ... 3.12) | -0.27  (-0.33 ... -0.19) | good |
| NESM3 (1) | good | good | 2.92  (2.54 ... 3.36) | -0.18  (-0.32 ... -0.04) | good |
| NorESM2-MM (1) | good | good | 3.05  (2.71 ... 3.49) | -0.34  (-0.44 ... -0.24) | bad (sigma too large; shape too small) |
| UKESM1-0-LL (5) | good | good | 2.89  (2.74 ... 3.06) | -0.18  (-0.2 ... -0.16) | good |

**Results and synthesis**

In this section we present the full breakdown by observational and model dataset of the results of the attribution analysis of section 5 of the main text. The results for the threshold used, PR and change in intensity for both 2020 and 2050 with respect to 1900 are first given for the Siberian region (Table S3, Figure S5) and then for Verkhoyansk (Table S4, Figure S6).

*Table S3: Results of the attribution analysis of the prolonged heat in the Siberian region, comparing the event to a 1900 climate, as well as comparing future events of similar magnitudes to a 1900 climate.*

| **Model / Observations** | **Threshold for return period 130 yr [°C]** | **Ypast - Ynow** | **Probability ratio PR** | **Change in intensity ΔI [°C]** | **Ypast - Yfuture** | **Probability ratio PR** | **Change in intensity ΔI [°C]** |
| --- | --- | --- | --- | --- | --- | --- | --- |
| ERA5 (fit to 1979 - 2019) | -8.759 °C | 1900 - 2020 | 77268000 (22495 ... 158670000000000) | 4.081 (2.417 ... 5.554) |  |  |  |
| GISTEMP 250km anomalies (fit to 1916 - 2019) | 5.7131 °C | 1900 - 2020 | 81976 (3581.6 ... 10550000) | 2.846 (2.142 ... 3.516) |  |  |  |
| MPI-ESM1.2-HR (10) | -8.383 | 1900 - 2020 | 5157  (2172.3 ... 10824) | 2.417  (2.192 ... 2.542) | 1900 - 2050 | 114610  (52172 ... 242490) | 3.913  (3.727 ... 4.081) |
| BCC_bcc-csm1-1-m | -7.75 | 1900 - 2020 | 109016.66  (37772.48 ... 408410.81) | 2.72  (2.57 ... 2.88) | 1900 - 2050 | 3328189.32  (987395.36 ... 14261378.66) | 4.35  (4.14 ... 4.56) |
| BNU_BNU-ESM | -12.67 | 1900 - 2020 | 349475471.83  (34086945.87 ... 6166280784.11) | 4.11  (3.94 ... 4.29) | 1900 - 2050 | 31275151496.14  (2710147277.79 ... 606075303240.33) | 6.99  (6.74 ... 7.23) |
| CMCC_CMCC-CESM | -13.3 | 1900 - 2020 | 475.85  (207.2 ... 1221.83) | 1.76  (1.62 ... 1.9) | 1900 - 2050 | 23692.74  (8637.52 ... 72287.7) | 3.92  (3.71 ... 4.13) |
| CMCC_CMCC-CMS | -12.01 | 1900 - 2020 | 1027.34  (461.94 ... 2622.24) | 1.96  (1.85 ... 2.08) | 1900 - 2050 | 55815.48  (21644.69 ... 172007.66) | 4.24  (4.05 ... 4.46) |
| CNRM-CERFACS_CNRM-CM5 | -13.55 | 1900 - 2020 | 6400.76  (4050.22 ... 11345.34) | 2.43  (2.36 ... 2.53) | 1900 - 2050 | 257456.1  (153370.76 ... 474742.24) | 4.47  (4.38 ... 4.58) |
| CSIRO-BOM_ACCESS1-0 | -9.78 | 1900 - 2020 | 3583.94  (1833.78 ... 9089.35) | 2.5  (2.38 ... 2.66) | 1900 - 2050 | 179537.85  (79407.21 ... 501784.59) | 4.96  (4.75 ... 5.19) |
| CSIRO-BOM_ACCESS1-3 | -8.45 | 1900 - 2020 | 1851.25  (889.12 ... 4469.25) | 2.05  (1.93 ... 2.18) | 1900 - 2050 | 128529.22  (55978.8 ... 339380.17) | 4.57  (4.37 ... 4.78) |
| FIO_FIO-ESM | -10.75 | 1900 - 2020 | 334.42  (201.15 ... 596.89) | 1.61  (1.54 ... 1.68) | 1900 - 2050 | 4761.13  (2493.98 ... 9548.91) | 2.77  (2.65 ... 2.89) |
| INM_inmcm4 | -9.48 | 1900 - 2020 | 859.4  (380.86 ... 2464.4) | 1.87  (1.74 ... 1.99) | 1900 - 2050 | 21620.28  (7916.15 ... 79429.64) | 3.43  (3.21 ... 3.63) |
| IPSL_IPSL-CM5A-LR | -8.52 | 1900 - 2020 | 229787.88  (108683.52 ... 548418.33) | 2.51  (2.44 ... 2.59) | 1900 - 2050 | 13319339.61  (5846617.82 ... 34725830.68) | 4.44  (4.34 ... 4.54) |
| IPSL_IPSL-CM5A-MR | -7.1 | 1900 - 2020 | 38099.22  (14740.45 ... 125396.22) | 2.18  (2.08 ... 2.29) | 1900 - 2050 | 1950219.5  (649609.24 ... 7397378.47) | 3.96  (3.79 ... 4.13) |
| MIROC_MIROC-ESM-CHEM | -9.89 | 1900 - 2020 | 376026794.01  (35097379.79 ... 9904336716.06) | 3.8  (3.66 ... 3.98) | 1900 - 2050 | 46974628761.99  (4163914971.46 ... 1276608970267.47) | 7.61  (7.39 ... 7.84) |
| MIROC_MIROC-ESM | -9.63 | 1900 - 2020 | 14690116.44  (2754617.4 ... 119119789.32) | 3.55  (3.4 ... 3.73) | 1900 - 2050 | 1643668973.54  (292485620.53 ... 13914772142.24) | 6.94  (6.72 ... 7.17) |
| MIROC_MIROC5 | -11.64 | 1900 - 2020 | 27914.24  (13811.83 ... 68475.76) | 2.42  (2.32 ... 2.55) | 1900 - 2050 | 1714170.06  (785785.91 ... 4483455.02) | 4.62  (4.49 ... 4.76) |
| MPI-M_MPI-ESM-MR | -8.24 | 1900 - 2020 | 77916.1  (29817.83 ... 269695.66) | 2.99  (2.86 ... 3.15) | 1900 - 2050 | 3094325.79  (1024242.05 ... 12206162.06) | 5.06  (4.85 ... 5.28) |
| MRI_MRI-CGCM3 | -10.55 | 1900 - 2020 | 128.06  (77.3 ... 241.85) | 1.65  (1.54 ... 1.78) | 1900 - 2050 | 3363.92  (1700.61 ... 7814.05) | 3.5  (3.27 ... 3.74) |
| NASA-GISS_GISS-E2-H-CC | -10.22 | 1900 - 2020 | 519844.65  (96020.82 ... 5191824.61) | 2.8  (2.64 ... 2.97) | 1900 - 2050 | 18876850.06  (2891642.34 ... 238869471.96) | 4.45  (4.21 ... 4.68) |
| NASA-GISS_GISS-E2-H | -9.78 | 1900 - 2020 | 432483.4  (176611.48 ... 1243253.83) | 2.61  (2.53 ... 2.73) | 1900 - 2050 | 19608930.67  (7265526.97 ... 60235112.47) | 4.34  (4.21 ... 4.47) |
| NASA-GISS_GISS-E2-R | -10.68 | 1900 - 2020 | 7697.5  (3934.54 ... 16458.92) | 1.99  (1.9 ... 2.09) | 1900 - 2050 | 114459.43  (52260.12 ... 278965.66) | 3.03  (2.89 ... 3.17) |
| NCAR_CCSM4 | -10.39 | 1900 - 2020 | 75048.62  (41686.78 ... 144698.82) | 3.15  (3.08 ... 3.26) | 1900 - 2050 | 2538659.66  (1322783.9 ... 5128333.79) | 5.18  (5.08 ... 5.29) |
| NCC_NorESM1-M | -11.8 | 1900 - 2020 | 5621.11  (2512.73 ... 15190.98) | 2.66  (2.52 ... 2.81) | 1900 - 2050 | 233163.6  (89455.08 ... 725074.14) | 4.94  (4.72 ... 5.16) |
| NSF-DOE-NCAR_CESM1-BGC | -9.93 | 1900 - 2020 | 23189.75  (7546.63 ... 109379.49) | 3.04  (2.87 ... 3.21) | 1900 - 2050 | 678296.23  (190159.62 ... 3872978.88) | 5.03  (4.77 ... 5.28) |
| HadGEM3-A ALL | -12.06 | 1900 - 2020 | 2361200  (146240 ... 68329000) | 3.41  (2.86 ... 4.06) | 1900 - 2050 | ( ... ) | ( ... ) |
| AWI-CM-1-1-MR (1) | -7.574 | 1900 - 2020 | 14030.35  (687.19 ... 439020) | 439020  (2.03 ... 3.44) | 1900 - 2050 | 3579577050  (21845129.1 ... 1221722830000) | 4.86  (4.26 ... 5.46) |
| BCC-CSM2-MR (1) | -9.962 | 1900 - 2020 | 471.1  (47.1 ... 5868.6) | 5868.6  (1.12 ... 2.4) | 1900 - 2050 | 282823902  (3075592.16 ... 48481837100) | 4.2  (3.63 ... 4.78) |
| CESM2 (5) | -7.59 | 1900 - 2020 | 63089.58  (14487.54 ... 315955.59) | 315955.59  (2.94 ... 3.59) | 1900 - 2050 | 11220208200  (1017963800 ... 138662239000) | 5.61  (5.31 ... 5.91) |
| CESM2-WACCM (3) | -9.024 | 1900 - 2020 | 16768.21  (3225.11 ... 103297.11) | 2.79  (2.42 ... 3.16) | 1900 - 2050 | 2635871170  (144754117 ... 57141801500) | 4.97  (4.61 ... 5.31) |
| CIESM (1) | -9.905 | 1900 - 2020 | 29.11  (3.11 ... 275.36) | 1.25  (275.36 ... 2.06) | 1900 - 2050 | 695.57  (68.71 ... 8193.69) | 2.15  (1.46 ... 2.83) |
| CMCC-CM2-SR5 (1) | -9.23 | 1900 - 2020 | 469151.97  (13776.54 ... 27819549.5) | 3.18  (2.63 ... 3.72) | 1900 - 2050 | 7806252650000000  (2379992540000 ... 102045113000000000000) | 6.62  (5.99 ... 7.23) |
| CNRM-CM6-1-HR (1) | -10.418 | 1900 - 2020 | 10098.68  (437.49 ... 315195.18) | 10098.68  (315195.18 ... 3.05) | 1900 - 2050 | 35443181600  (151606194 ... 25362255800000) | 4.87  (4.25 ... 5.48) |
| CanESM5 (50) | -7.432 | 1900 - 2020 | 134473.15  (79965.55 ... 223437.51) | 134473.15  (223437.51 ... 3.52) | 1900 - 2050 | 155126591000000  (53282991600000 ... 449637340000000) | 6.87  (6.78 ... 6.96) |
| CanESM5-CanOE (3) | -7.72 | 1900 - 2020 | 104703.24  (13061.39 ... 1025559.75) | 3.32  (2.83 ... 3.78) | 1900 - 2050 | 138593564000000  (1968567690000 ... 14436250900000000) | 6.77  (6.39 ... 7.18) |
| EC-Earth3 (7) | -11.092 | 1900 - 2020 | 864766.8  (205824.01 ... 3759569.21) | 864766.8  (3759569.21 ... 3.5) | 1900 - 2050 | 274833638000000  (15687565500000 ... 5837131710000000) | 6.09  (5.87 ... 6.31) |
| EC-Earth3-Veg (4) | -10.87 | 1900 - 2020 | 3323104.59  (484670.15 ... 28209229.96) | 3.28  (3.04 ... 3.53) | 3.53 - 2050 | 657934104000000  (14412277500000 ... 39088882000000000) | 5.98  (5.73 ... 6.24) |
| FGOALS-f3-L (1) | -12.515 | 1900 - 2020 | 1287.81  (93.35 ... 23213.61) | 1287.81  (23213.61 ... 3.09) | 3.09 - 2050 | 991540124  (7443665.77 ... 205393237000) | 5.14  (4.46 ... 5.84) |
| FGOALS-g3 (4) | -13.724 | 1900 - 2020 | 36972.49  (7296.8 ... 200192.58) | 2.83  (2.51 ... 3.15) | 1900 - 2050 | 413274996  (41524226.4 ... 4855558040) | 4.4  (4.12 ... 4.68) |
| FIO-ESM-2-0 (3) | -10.406 | 1900 - 2020 | 105821.55  (13287.24 ... 989541.44) | 105821.55  (989541.44 ... 3.87) | 1900 - 2050 | 2955730750  (166029062 ... 72304549500) | 5.45  (5.04 ... 5.86) |
| GFDL-CM4 (1) | -10.953 | 1900 - 2020 | 279167.26  (8047.34 ... 14790299.7) | 2.82  (2.23 ... 3.42) | 1900 - 2050 | 4044132030000000  (1891764020000 ... 27850197600000000000) | 5.98  (5.44 ... 6.54) |
| GFDL-ESM4 (1) | -9.868 | 1900 - 2020 | 21.62  (3.71 ... 120.94) | 21.62  (120.94 ... 1.24) | 1900 - 2050 | 878680.05  (33238.12 ... 35559254.2) | 2.53  (2.11 ... 2.94) |
| HadGEM3-GC31-LL (4) | -11.489 | 1900 - 2020 | 37360.52  (7614 ... 208395.23) | 37360.52  (208395.23 ... 2.74) | 1900 - 2050 | 23630039500000  (791620476000 ... 946091201000000) | 5.38  (5.12 ... 5.65) |
| HadGEM3-GC31-MM (3) | -11.466 | 1900 - 2020 | 308.05  (81.62 ... 1127.66) | 1.55  (1.21 ... 1.89) | 1900 - 2050 | 107727278000  (4009022080 ... 3653994640000) | 4.69  (4.37 ... 5.01) |
| INM-CM4-8 (1) | -11.076 | 1900 - 2020 | 71.8  (8.47 ... 667.34) | 71.8  (667.34 ... 1.63) | 1900 - 2050 | 24064298.1  (417335.6 ... 2751496950) | 3.16  (2.68 ... 3.63) |
| IPSL-CM6A-LR (6) | -8.987 | 1900 - 2020 | 33851.79  (8945.43 ... 140497.11) | 33851.79  (140497.11 ... 3.02) | 1900 - 2050 | 4588690190000  (298324221000 ... 84410690100000) | 5.61  (5.36 ... 5.86) |
| MIROC6 (50) | -9.492 | 1900 - 2020 | 12671  (8277.73 ... 19659.39) | 12671  (19659.39 ... 2.56) | 1900 - 2050 | 1264371450000  (503891431000 ... 3224805860000) | 4.8  (4.72 ... 4.87) |
| MPI-ESM1-2-HR (2) | -8.523 | 1900 - 2020 | 2097.08  (285.84 ... 17477.45) | 2.28  (1.79 ... 2.26) | 1900 - 2050 | 10047945.1  (559578.91 ... 247646638) | 3.86  (3.41 ... 4.3) |
| MPI-ESM1-2-LR (10) | -9.814 | 1900 - 2020 | 6198.4  (2432.36 ... 15973.56) | 6198.4  (15973.56 ... 2.78) | 1900 - 2050 | 5128678180  (951888987 ... 28953417500) | 4.73  (4.54 ... 4.92) |
| NorESM2-MM (1) | -11.012 | 1900 - 2020 | 122.63  (21.64 ... 786.05) | 1.66  (1.09 ... 2.98) | 1900 - 2050 | 435810.3  (18596.74 ... 15189481.98) | 3.75  (3.1 ... 4.37) |
| UKESM1-0-LL (5) | -12.621 | 1900 - 2020 | 5774.56  (1698.12 ... 21228.96) | 5774.56  (21228.96 ... 2.2) | 1900 - 2050 | 561283720000000000  (9465296850000000 ... 45007401000000000000) | 6.81  (6.56 ... 7.06) |
| MPI-GE (100) | -8.572 | 1900 - 2020 | 26073  (20296 ... 31948) | 2.858  (2.792 ... 2.862) | 1900 - 2050 | 1444500  (1046300 ... 1828700) | 4.991  (4.927 ... 4.994) |

*Table S4: Results of the attribution analysis of the record-breaking high June temperature at Verkhoyansk station, comparing the event to a 1900 climate, as well as comparing future events of similar magnitudes to a 1900 climate.*

| **Model / Observations** | **Threshold for return period 140 yr** | **Ypast - Ynow** | **Probability ratio PR** | **Change in intensity ΔI** | **Ypast - Yfuture** | **Probability ratio PR** | **Change in intensity ΔI** |
| --- | --- | --- | --- | --- | --- | --- | --- |
| Verkhoyansk (fit to 1926 - 2019) | 38 °C | 1900 - 2020 | undefined (2.8055 ... undefined) | 1.041 (0.352 ... 3.368) |  |  |  |
| Verkhoyansk (fit to 1926 - 2020) (MF method) | 38 °C | 1900 - 2020 | inf (7.29 ... inf) | 1.63 (1 ... 2.29) |  |  |  |
| CMCC_CMCC-CMS | 25.69 | 1900 - 2020 | 18.68  (4.75 ... inf) | 1.26  (0.99 ... 1.52) | 1900 - 2050 | 104.17  (17.36 ... inf) | 2.72  (2.16 ... 3.28) |
| CNRM-CERFACS_CNRM-CM5 | 34.05 | 1900 - 2020 | 7.75  (4.67 ... 15.06) | 1.3  (1.06 ... 1.54) | 1900 - 2050 | 25.57  (12.73 ... 60.31) | 2.39  (1.95 ... 2.82) |
| CSIRO-BOM_ACCESS1-0 | 34.95 | 1900 - 2020 | 10.42  (4.48 ... 83.61) | 1.35  (1.1 ... 1.62) | 1900 - 2050 | 44.29  (13.82 ... 501.33) | 2.67  (2.19 ... 3.19) |
| CSIRO-BOM_ACCESS1-3 | 29.46 | 1900 - 2020 | 14.42  (5.59 ... inf) | 1.06  (0.85 ... 1.31) | 1900 - 2050 | 85.71  (24.03 ... inf) | 2.38  (1.91 ... 2.9) |
| CSIRO-QCCCE_CSIRO-Mk3-6-0 | 27.6 | 1900 - 2020 | 7.51  (5.45 ... 11.75) | 1.08  (1 ... 1.17) | 1900 - 2050 | 33.29  (21.57 ... 59.14) | 2.32  (2.16 ... 2.47) |
| IPSL_IPSL-CM5A-LR | 28.2 | 1900 - 2020 | inf  (1239.07 ... inf) | 2.08  (1.96 ... 2.21) | 1900 - 2050 | inf  (10113.92 ... inf) | 3.68  (3.48 ... 3.89) |
| IPSL_IPSL-CM5B-LR | 28.94 | 1900 - 2020 | inf  (146.73 ... inf) | 1.67  (1.46 ... 1.9) | 1900 - 2050 | inf  (855.31 ... inf) | 2.94  (2.56 ... 3.34) |
| MRI_MRI-CGCM3 | 35.71 | 1900 - 2020 | 3.76  (2.36 ... 8.39) | 0.97  (0.7 ... 1.21) | 1900 - 2050 | 11.42  (5.29 ... 36.26) | 2.05  (1.48 ... 2.57) |
| NASA-GISS_GISS-E2-H | 33.33 | 1900 - 2020 | 19.42  (6.24 ... inf) | 1.89  (1.59 ... 2.18) | 1900 - 2050 | 70.75  (17.07 ... inf) | 3.13  (2.62 ... 3.63) |
| NASA-GISS_GISS-E2-R | 32.85 | 1900 - 2020 | 10.07  (4.39 ... 74.74) | 1.74  (1.39 ... 2.07) | 1900 - 2050 | 25.18  (8.85 ... 261.42) | 2.65  (2.12 ... 3.15) |
| HadGEM3-A | 30.819 | 1900 - 2020 | 103000000  (132.02 ... inf) | 3.272  (2.178 ... 4.17) | 1900 - 2050 | ( ... ) | ( ... ) |
| ACCESS-CM2 (2) | 25.772 | 1900 - 2020 | inf  (11.11 ... inf) | 1.98  (0.83 ... 3.12) | 1900 - 2050 | inf  (inf ... inf) | ( ... ) |
| ACCESS-ESM1-5 (3) | 27.13 | 1900 - 2020 | inf  (16.78 ... inf) | 2.02  (1.23 ... 2.81) | 1900 - 2050 | inf  (491.56 ... inf) | ( ... ) |
| AWI-CM-1-1-MR (1) | 37.406 | 1900 - 2020 | inf  (9.29 ... inf) | 3.8  (1.56 ... 5.87) | 1900 - 2050 | inf  (14.66 ... inf) | ( ... ) |
| CMCC-CM2-SR5 (1) | 27.939 | 1900 - 2020 | inf  (9.67 ... inf) | 3.71  (2.31 ... 5.09) | 1900 - 2050 | inf  (118.11 ... inf) | ( ... ) |
| CNRM-CM6-1 (1) | 28.596 | 1900 - 2020 | 6.19  (0.6 ... inf) | 0.94  (-0.21 ... 2) | 1900 - 2050 | inf  (16.15 ... inf) | ( ... ) |
| CNRM-CM6-1-HR (1) | 31.628 | 1900 - 2020 | 28.61  (0.97 ... inf) | 1.6  (-0.03 ... 3.26) | 1900 - 2050 | inf  (11.74 ... inf) | ( ... ) |
| CNRM-ESM2-1 (1) | 31.56 | 1900 - 2020 | 7.13  (1.86 ... inf) | 1.72  (0.58 ... 2.79) | 1900 - 2050 | 10.38  (2.02 ... inf) | ( ... ) |
| CanESM5 (50) | 27.837 | 1900 - 2020 | inf  (inf ... inf) | 3.35  (3.11 ... 3.6) | 1900 - 2050 | inf  (inf ... inf) | ( ... ) |
| EC-Earth3 (3) | 30.096 | 1900 - 2020 | inf  (120.51 ... inf) | 2.98  (1.78 ... 4.19) | 1900 - 2050 | inf  (inf ... inf) | ( ... ) |
| FGOALS-g3 (3) | 35.845 | 1900 - 2020 | 2795.4  (15.04 ... inf) | 2.24  (1.34 ... 3.24) | 1900 - 2050 | inf  (132.72 ... inf) | ( ... ) |
| GFDL-CM4 (1) | 29.478 | 1900 - 2020 | 6.81  (0.35 ... inf) | 1.2  (-0.56 ... 2.94) | 1900 - 2050 | 1.67  (0.28 ... 838234.7) | ( ... ) |
| GFDL-ESM4 (1) | 25.115 | 1900 - 2020 | 6.21  (0 ... inf) | 0.52  (-0.68 ... 1.84) | 1900 - 2050 | inf  (520.88 ... inf) | ( ... ) |
| HadGEM3-GC31-LL (4) | 28.65 | 1900 - 2020 | 946.91  (14.26 ... inf) | 2.33  (1.47 ... 3.13) | 1900 - 2050 | inf  (inf ... inf) | ( ... ) |
| HadGEM3-GC31-MM (3) | 33.144 | 1900 - 2020 | 2.51  (1.4 ... 4.72) | 1.44  (0.54 ... 2.34) | 1900 - 2050 | 32.43  (11.28 ... 133.27) | ( ... ) |
| INM-CM5-0 (1) | 28.265 | 1900 - 2020 | inf  (0.55 ... inf) | 1.49  (-0.2 ... 3.21) | 1900 - 2050 | inf  (713.31 ... inf) | ( ... ) |
| IPSL-CM6A-LR (6) | 31.568 | 1900 - 2020 | 6.71  (2.1 ... 61.87) | 1.27  (0.52 ... 1.99) | 1900 - 2050 | 548017350  (97.91 ... inf) | ( ... ) |
| MIROC6 (50) | 29.932 | 1900 - 2020 | 22.24  (11.84 ... 42.21) | 1.16  (0.94 ... 1.36) | 1900 - 2050 | inf  (inf ... inf) | ( ... ) |
| MPI-ESM1-2-HR (2) | 36.249 | 1900 - 2020 | 14.13  (1.73 ... inf) | 2.06  (0.49 ... 3.56) | 1900 - 2050 | 9.18  (1.62 ... 589.3) | ( ... ) |
| MPI-ESM1-2-LR (10) | 28.727 | 1900 - 2020 | 336.74  (17.58 ... inf) | 1.87  (1.28 ... 2.46) | 1900 - 2050 | inf  (inf ... inf) | ( ... ) |
| MRI-ESM2-0 (2) | 36.307 | 1900 - 2020 | 25.16  (1.87 ... inf) | 1.62  (0.37 ... 2.83) | 1900 - 2050 | inf  (51.61 ... inf) | ( ... ) |
| NESM3 (1) | 26.558 | 1900 - 2020 | 15.34  (1.48 ... inf) | 2.56  (0.31 ... 4.68) | 1900 - 2050 | 3123.42  (7.63 ... inf) | ( ... ) |
| UKESM1-0-LL (5) | 28.97 | 1900 - 2020 | 6.18  (2.64 ... 15.39) | 1.81  (0.98 ... 2.62) | 1900 - 2050 | 2645235120000  (5348.56 ... inf) | ( ... ) |

**
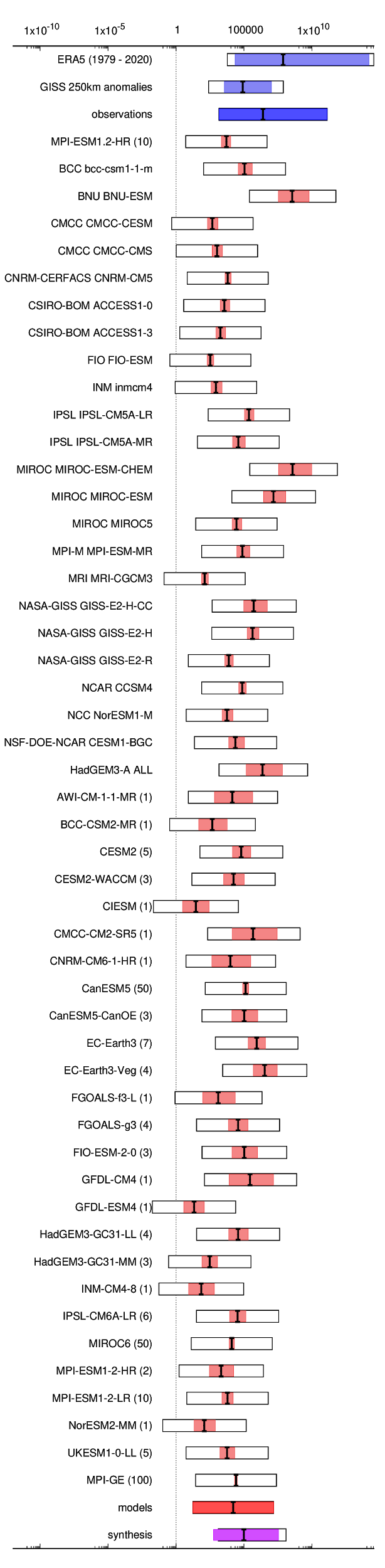
***
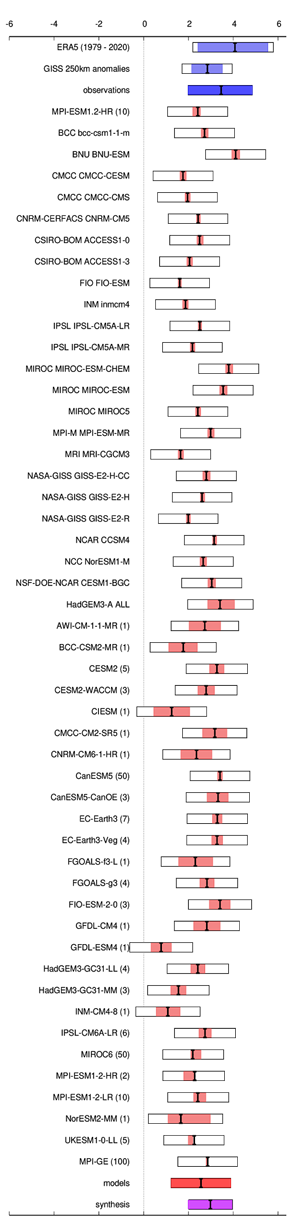
*

*Figure S5: Synthesis of probability ratios (left) and changes in intensity (right) from the attribution analysis of Jan-Jun mean temperature in Siberian region, comparing the 2020 event with 1900 climate. Date range for ERA5 expresses full length of data available.*

**
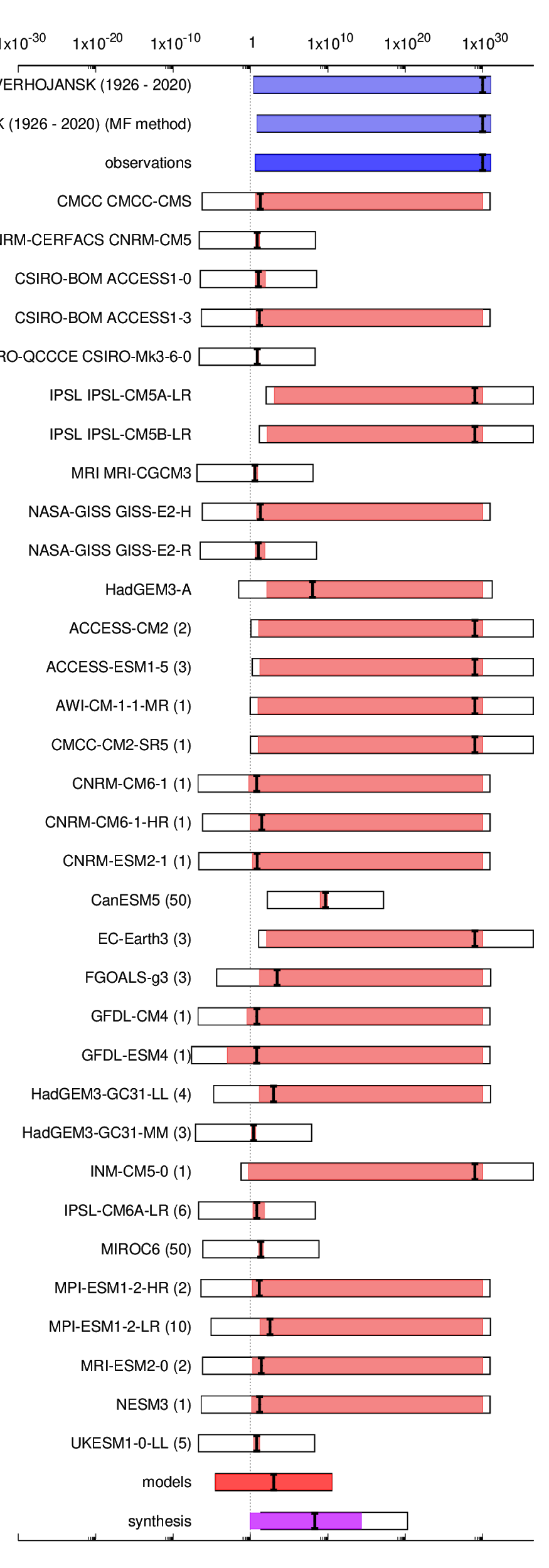

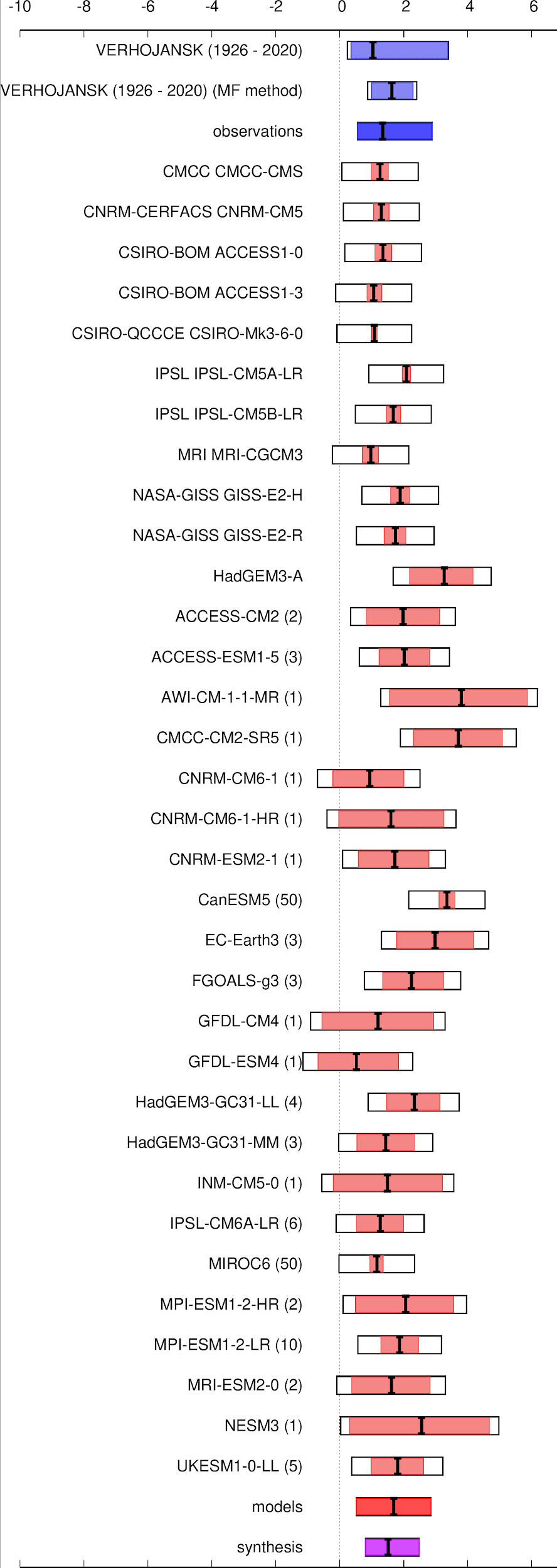
**

*Figure S6: Synthesis of probability ratios (left) and changes in intensity (right) from the attribution analysis of June TXx at Verkhoyansk station. Date ranges express full length of data available for analysis.*

**SMILEs analysis results**

This section presents the PR results from the parallel analysis of SMILEs large ensembles broken down by model for the Siberian region (Table S5) and for Verkhoyansk (Table S6). These are also summarised as bar plots (Figure S10).

*Table S5: SMILEs PR results for the Siberian region.*

| **Dataset** | **RR 2020 vs 1950** | | | **RR 2050 vs 1950** | | |
| --- | --- | --- | --- | --- | --- | --- |
|  | **Best estimate** | **Lower bound** | Upper bound | **Best estimate** | **Lower bound** | Upper bound |
| MPI-ESM | 1486.4 | 224.9 | 15718.0 | 49441.0 | 6767.2 | 497080.0 |
| CESM1-CAM5 | 1996.3 | 227.6 | 66585.0 | 93916.0 | 10201.0 | 3396900.0 |
| CSIRO-Mk3-6-0 | 3849.3 | 31.3 | 61433000.0 | 287320.0 | 2412.0 | 4675600000.0 |
| CanESM2 | 2401.4 | 148.1 | 229180.0 | 39400.0 | 2349.2 | 3555100.0 |
| EC-EARTH | 22331.0 | 145.1 | 7401900000.0 | 297010.0 | 2033.8 | 98045000000.0 |
| GFDL-ESM2M | 2233.5 | 157.6 | 116050.0 | 31075.0 | 2082.5 | 1531600.0 |
| GFDL-CM3 | 2479.5 | 52.8 | 6601500.0 | 288640.0 | 6148.0 | 766010000.0 |

*Table S6: SMILEs PR results for Verkhoyansk.*

| **Dataset** | **RR 2020 vs 1950** | | |
| --- | --- | --- | --- |
|  | **Best estimate** | **Lower bound** | **Upper bound** |
| CESM1-CAM5 | 27.2 | 2.0 | Inf |
| CSIRO-Mk3-6-0 | 1.3 | 0.3 | Inf |
| CanESM2 | 12.0 | 0.7 | Inf |
| EC-EARTH | 1.7 | 0.3 | Inf |
| GFDL-ESM2M | 2.6 | 0.4 | Inf |
| GFDL-CM3 | 0.6 | 0.2 | Inf |


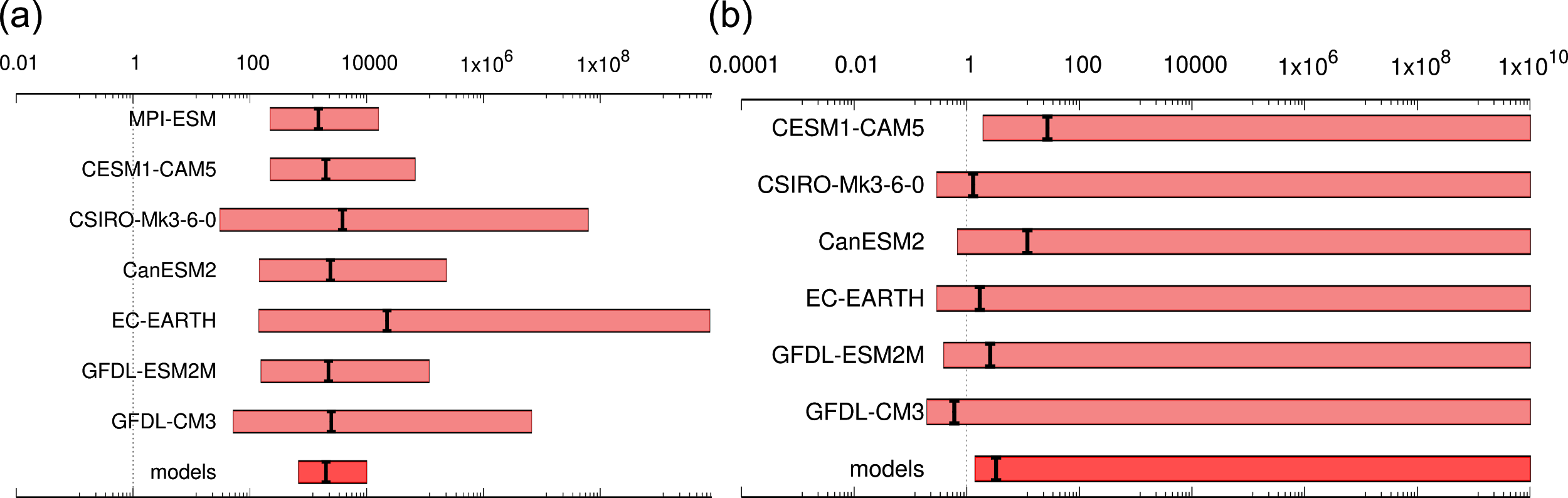


*Figure S7: SMILEs large ensemble CMIP5 results for PR in the year 2020 for the Siberian region (a) and Verkhoyansk (b). A similar picture to the results of the main method of analysis is found: in the Siberian region the PR is confidently large while for Verkhoyansk there is much lower confidence but the PR may be very large (1950 baseline used instead of 1900, lowering the PR).*

**Future model results for main analysis**

This section presents the breakdown by model of the results for PR and intensity in the year 2050 using the main method of analysis of section 5 of the main text. Figure S8 presents these results for the Siberian region and for obvious reasons contains only values from model projections so that no synthesis with observations is presented.

*
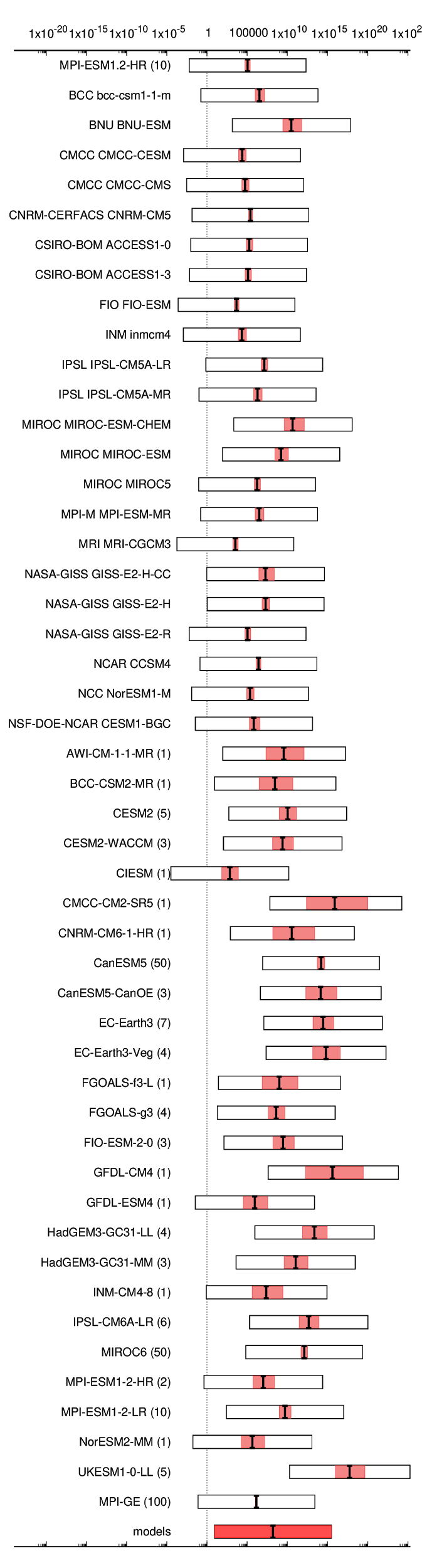

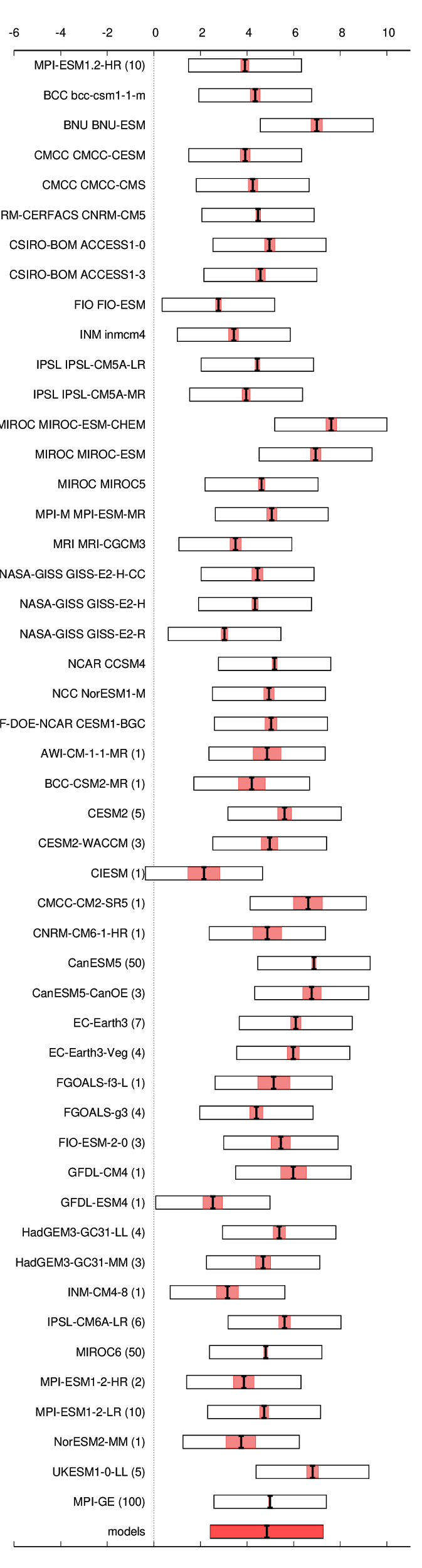
*

*Figure S8: Synthesis of probability ratios (left) and changes in intensity (right) from the attribution analysis of Jan-Jun mean temperature in Siberian region, comparing future 2050 climate with 1900 climate.*
